# Trans‐species synthetic gene design allows resistance pyramiding and broad‐spectrum engineering of virus resistance in plants

**DOI:** 10.1111/pbi.12896

**Published:** 2018-03-05

**Authors:** Anna Bastet, Baptiste Lederer, Nathalie Giovinazzo, Xavier Arnoux, Sylvie German‐Retana, Catherine Reinbold, Véronique Brault, Damien Garcia, Samia Djennane, Sophie Gersch, Olivier Lemaire, Christophe Robaglia, Jean‐Luc Gallois

**Affiliations:** ^1^ GAFL INRA Montfavet France; ^2^ Aix Marseille University UMR 7265 Biologie Végétale et Microbiologie Environnementales Laboratoire de Génétique et Biophysique des Plantes Marseille France; ^3^ CNRS UMR 7265 Biologie Végétale et Microbiologie Environnementales Marseille France; ^4^ CEA Bioscience and Biotechnology Institute of Aix‐Marseille Marseille France; ^5^ UMR 1332 Biologie du Fruit et Pathologie INRA Univ. Bordeaux Villenave d'Ornon France; ^6^ Université de Strasbourg INRA UMR‐A 1131 Santé de la Vigne et Qualité du Vin Colmar France; ^7^ Centre National de la Recherche Scientifique Institut de Biologie Moléculaire des Plantes (IBMP) UPR 2357 Strasbourg France

**Keywords:** eIF4E, potyvirus, resistance, Arabidopsis thaliana, synthetic allele, translational research

## Abstract

To infect plants, viruses rely heavily on their host's machinery. Plant genetic resistances based on host factor modifications can be found among existing natural variability and are widely used for some but not all crops. While biotechnology can supply for the lack of natural resistance alleles, new strategies need to be developed to increase resistance spectra and durability without impairing plant development. Here, we assess how the targeted allele modification of the *Arabidopsis thaliana* translation initiation factor eIF4E1 can lead to broad and efficient resistance to the major group of potyviruses. A synthetic *Arabidopsis thaliana eIF4E1* allele was designed by introducing multiple amino acid changes associated with resistance to potyvirus in naturally occurring *Pisum sativum* alleles. This new allele encodes a functional protein while maintaining plant resistance to a potyvirus isolate that usually hijacks eIF4E1. Due to its biological functionality, this synthetic allele allows, at no developmental cost, the pyramiding of resistances to potyviruses that selectively use the two major translation initiation factors, eIF4E1 or its isoform eIFiso4E. Moreover, this combination extends the resistance spectrum to potyvirus isolates for which no efficient resistance has so far been found, including resistance‐breaking isolates and an unrelated virus belonging to the *Luteoviridae* family. This study is a proof‐of‐concept for the efficiency of gene engineering combined with knowledge of natural variation to generate trans‐species virus resistance at no developmental cost to the plant. This has implications for breeding of crops with broad‐spectrum and high durability resistance using recent genome editing techniques.

## Introduction

Synthetic biology has emerged as a promising tool to provide advantageous new functions for crop improvement using plant biotechnology (Liu and Stewart, 2015). One aspect of synthetic biology is the possibility of editing the plant's own genes to induce changes in the way the plant interacts with its environment (Baltes and Voytas, 2015). This approach was successfully used to improve water use and drought stress tolerance in tomato (*Solanum lycopersicum*) and *Arabidopsis thaliana* by modifying the abscisic acid (ABA) receptor PYRABACTIN RESISTANCE 1 to make it responsive to an agrochemical ligand (Park *et al*., 2015). Resistance to pathogens is also an important field in which gene engineering can play a major role by restricting the way pathogens hijack host factors essential for their infectious cycle. For example, targeted modification of the JA hormone receptor in *Arabidopsis* maintains efficient resistance to the bacterial effector from *Pseudomonas syringae* without impairing the JA recognition pathway (Zhang *et al*., 2015). It is of particular interest to understand how specific modifications of plant genes, often resulting from nonsynonymous Amino Acid (AA) changes, can generate new functions. These modifications can then be implemented through high‐throughput allele mining (Barabaschi *et al*., 2016) or new genome editing technologies such as CRISPR/Cas9 (Ma *et al*., 2016). Overall, gene editing technologies appear as a promising tool to enhance and accelerate plant breeding (Østerberg *et al*., 2017; Palmgren *et al*., 2015).

Viruses are obligate intracellular parasites encoding very few proteins and are thus highly dependent on host factors for successful infection. A widely developed strategy to counter viral infections in crops is to modify these factors or inhibit their expression, therefore causing resistance by loss‐of‐susceptibility (Pavan *et al*., 2010; van Schie and Takken, 2014). The eukaryotic initiation factor 4E (eIF4E) and its isoform (eIFiso4E) have been proven to be susceptibility factors to several economically important single‐stranded positive sense RNA (ssRNA+) viruses (mainly *Potyviridae* family members) in many crops (Robaglia and Caranta, 2006; Wang and Krishnaswamy, 2012). In cells, translation initiation factors 4E are essential elements of the cellular translation initiation process and are in charge of binding the capped mRNA and recruiting the other translation initiation factors (Browning and Bailey‐Serres, 2015).

Natural diversity is a great reservoir of *eIF4E* resistance alleles in crops and their related wild species. Mostly, these resistance alleles encode modified eIF4E proteins, which still retain a functional role in translation initiation while carrying nonsynonymous AA changes, often located in two conserved regions of the protein (Robaglia and Caranta, 2006). Such resistance alleles have been successfully deployed in many crop species such as tomato, barley (*Hordeum vulgare)*, lettuce (*Lactuca sativa*), melon (*Cucumis melo*) and pepper (*Capsicum annuum)* (Nicaise *et al*., 2003; Nieto *et al*., 2006; Ruffel *et al*., 2002, 2005; Stein *et al*., 2005). However, such natural virus resistance alleles are not available in some other important crops such as papaya *(Carica papaya)*,* Prunus* species or cassava (*Manihot esculenta)*, which are challenged by economically important viruses such as *Papaya ringspot virus*,* Plum pox virus* and *Cassava brown streak virus,* respectively (Bart and Taylor, 2017; García *et al*., 2014; Gonsalves, 1998). Moreover, even if resistance alleles are present in related wild species, their introgression into cultivated accessions can be difficult in the case of reproductive incompatibility or genetic linkage drag bringing unwanted traits (Lin *et al*., 2014). To compensate for this lack of natural alleles, *eIF4E* gene disruption can be used to induce virus resistance. Such approaches have been successfully used in some species such as Arabidopsis and cucumber (*Cucumis sativus* L.) (Chandrasekaran *et al*., 2016; Duprat *et al*., 2002; Pyott *et al*., 2016). Because of redundancy among the *eIF4E* gene family, plants knocked out (KO) for a single translation initiation factor mostly exhibit normal physiology (Bastet *et al*., 2017). However, as ssRNA^+^ viruses recruit selectively distinct members of the *eIF4E* gene family, a single KO is often associated with a limited resistance spectrum while simultaneous KO of several members of the *eIF4E* gene family often leads to lethality or impaired growth (Callot and Gallois, 2014; Gauffier *et al*., 2016). Consequently, pyramiding resistances by knocking out several factors to extend the virus resistance spectrum can be impeded by the induction of developmental defects as shown in *Arabidopsis* (Callot and Gallois, 2014) and tomato (Gauffier *et al*., 2016). It is also expected that redundancy among 4E (*i.e. eIF4E* or *eIFiso4E*) genes could reduce eIF4E‐based resistance durability by making other 4E factors available to viruses (Bastet *et al*., 2017). Finally, a comparison between natural functional *eIF4E* resistance alleles and engineered loss‐of‐function KO alleles in tomato unveiled unexpected regulatory processes between the members of the eIF4E family making this approach less effective (Gauffier *et al*., 2016).

In the light of these observations, the best strategy to develop eIF4E‐based resistance would be to design functional alleles by introducing point mutations in the gene, mimicking naturally occurring resistances, instead of knocking them out (Bastet *et al*., 2017). In this regard, several studies have shown that *eIF4E* or *eIFiso4E* could be engineered as resistance alleles, in tomato, potato (*Solanum tuberosum)* and Chinese cabbage (*Brassica rapa)*, as attested by the transgenic ectopic expression of resistance alleles under the control of a strong constitutive promoter in a susceptible genetic background (Cavatorta *et al*., 2011; Kang *et al*., 2007; Kim *et al*., 2014).

In the present paper, we aimed at extending this approach by mimicking allele replacement and addressing the functional role of the modified eIF4E protein using the well‐characterized *Arabidopsis thaliana/potyvirus* pathosystem (Ouibrahim and Caranta, 2013).

We report for the first time the construction of a synthetic resistance allele of *eIF4E1* by introducing six nonsynonymous mutations deduced from pea *eIF4E* allelic variability. We show that a phenotypic defect associated with *eif4e1* loss of function, a bolting delay, can be complemented by the synthetic allele, while maintaining the resistance to the potyvirus *Clover yellow vein virus* (ClYVV). Significantly, by maintaining function *in planta*, this synthetic resistance allele allows resistance pyramiding with an *eifiso4e* loss‐of‐function allele and confers new resistances to another important potyvirus species as well as to previously described *eIFiso4E*‐resistance‐breaking (RB) isolates. In addition, these double‐mutated plants displayed resistance to a polerovirus species. This study therefore establishes a proof‐of‐concept for editing susceptibility genes to create new genetic resistances and expand resistance spectrum without affecting the plant development and agronomic traits.

## Results

### Design of a synthetic *eIF4E1* ^*R*^ resistance allele in Arabidopsis

**Figure 1:**
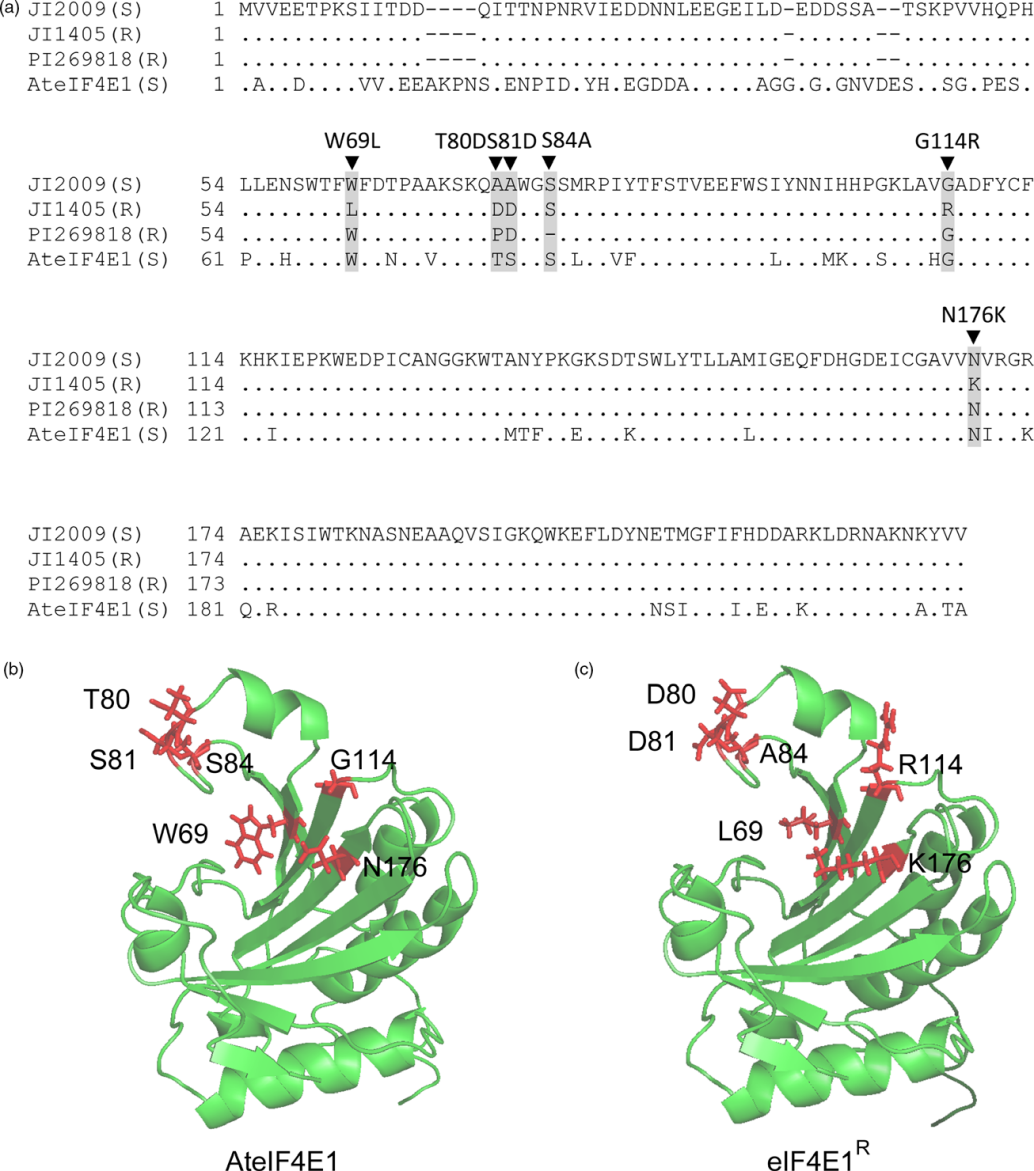
Design of a synthetic *Arabidopsis* resistance allele *eIF4E1* ^*R*^. (a) Alignment of eIF4E protein sequences from pea susceptible (JI2009) and resistant (JI1405 and PI269818) accessions with the *Arabidopsis thaliana* eIF4E1 (AteIF4E1). The position of the amino acids that differ between susceptible and resistance pea accessions is highlighted in grey. Black triangles above the alignment indicate the mutations introduced in *Arabidopsis* eIF4E1 to create the synthetic eIF4E1^R^. (b and c) Three‐dimensional predicted structure of AteIF4E1 (b) and of eIF4E1^R^ (c) based on homology modelling using the pea eIF4E 3D structure as a template (PDB ID: 2WMC‐C). The modified amino acids between AteIF4E1 and eIF4E1^R^ are coloured in red with side chains shown.

The wild‐type *Arabidopsis eIF4E1* gene from the Columbia (Col) accession was chosen as a target to design a synthetic resistance allele. *eIF4E1* encodes a susceptibility factor to the potyvirus *Clover yellow vein virus* (ClYVV) and, consequently, inactivation of *eIF4E1* (*eif4e1* ^*KO*^) is associated with resistance to ClYVV (Sato *et al*., 2005). No natural *eIF4E1* resistance allele is known in *Arabidopsis*, so we looked for data on genetic resistance determinants in other species. We focused on *eIF4E1* natural variation in pea (*Pisum sativum*), because the natural *eIF4E* resistance allele (known as *sbm‐1, cyv‐2* or *wlv*) from pea accession JI1405 confers resistance to several potyviruses, notably ClYVV (Andrade *et al*., 2009; Bruun‐Rasmussen *et al*., 2007; Gao *et al*., 2004). The eIF4E1 protein in *Arabidopsis* is highly similar to its counterpart in pea (81% homology/89% similarity) (Gao *et al*., 2004) (Figure 1a). Another resistance allele isolated from pea accession PI269818 and conferring resistance to one pathotype of *Pea seed‐borne mosaic virus* (PsbMV) was also considered in this study. These two pea resistance alleles are associated with the presence of five AA substitutions and one deletion within the protein sequence: W62L (*i.e*. the tryptophan at position 62 is substituted by a leucine in the protein encoded by the resistance allele), A73D, A74D, G107R and N169K for JI1405 line, as well as the deleted serine at position 77 (S77) in the PI269818 accession. These AA changes are mainly located in two regions (regions I and II) close to the cap‐binding pocket according to the eIF4E 3D structure (Marcotrigiano *et al*., 1997; Robaglia and Caranta, 2006) (Figure S1). In *Arabidopsis*, the AA located at similar positions (AA 69, 80, 81, 114 and 176) are, in most cases, identical to the AA in the pea susceptible allele (Figure 1a). The synthetic *Arabidopsis eIF4E1* allele was obtained by substituting the AA present in the five locations by the corresponding AA from the resistant pea accession JI1405. The position of the deletion in the resistant pea accession PI269818 was substituted by an alanine in the *Arabidopsis* eIF4E1 sequence. The 3D predicted structure of the *Arabidopsis* eIF4E1 protein carrying the six modifications W69L, T80D, S81D, S84A, G114R, and N176K mimics the structure of the pea resistant alleles. No drastic changes in the protein structure were predicted, hinting that the function of this mutated form of eIF4E1 in translation initiation should not be strongly affected (Figure 1b,c and Figure S1).

All six nonsynonymous substitutions were introduced by PCR‐based directed mutagenesis on a 3.4‐kb construct covering the whole *Arabidopsis eIF4E1* gene including its endogenous promoter. This allele (called hereafter *eIF4E1* ^*R*^ *)* was introduced into *Arabidopsis* plants knocked out for *eIF4E1* (*eif4e1* ^*KO*^ lines), and three independent transgenic lines were selected for further analyses. The correct expression of the *eIF4E1* ^*R*^ transgene was checked in all three lines (Figure S2), as well as the sequence of the expressed *eIF4E1* mRNA in all lines. As controls, a construct harbouring a wild‐type *eIF4E1* was introduced into the same *eif4e1* ^*KO*^ background, as well as an unrelated transgene, expressing the GUS reporter gene under control of the CaMV 35S promoter.

### *eIF4E1* ^*R*^ encodes a functional protein that complements *eif4e1* ^*KO*^ bolting delay

**Figure 2:**
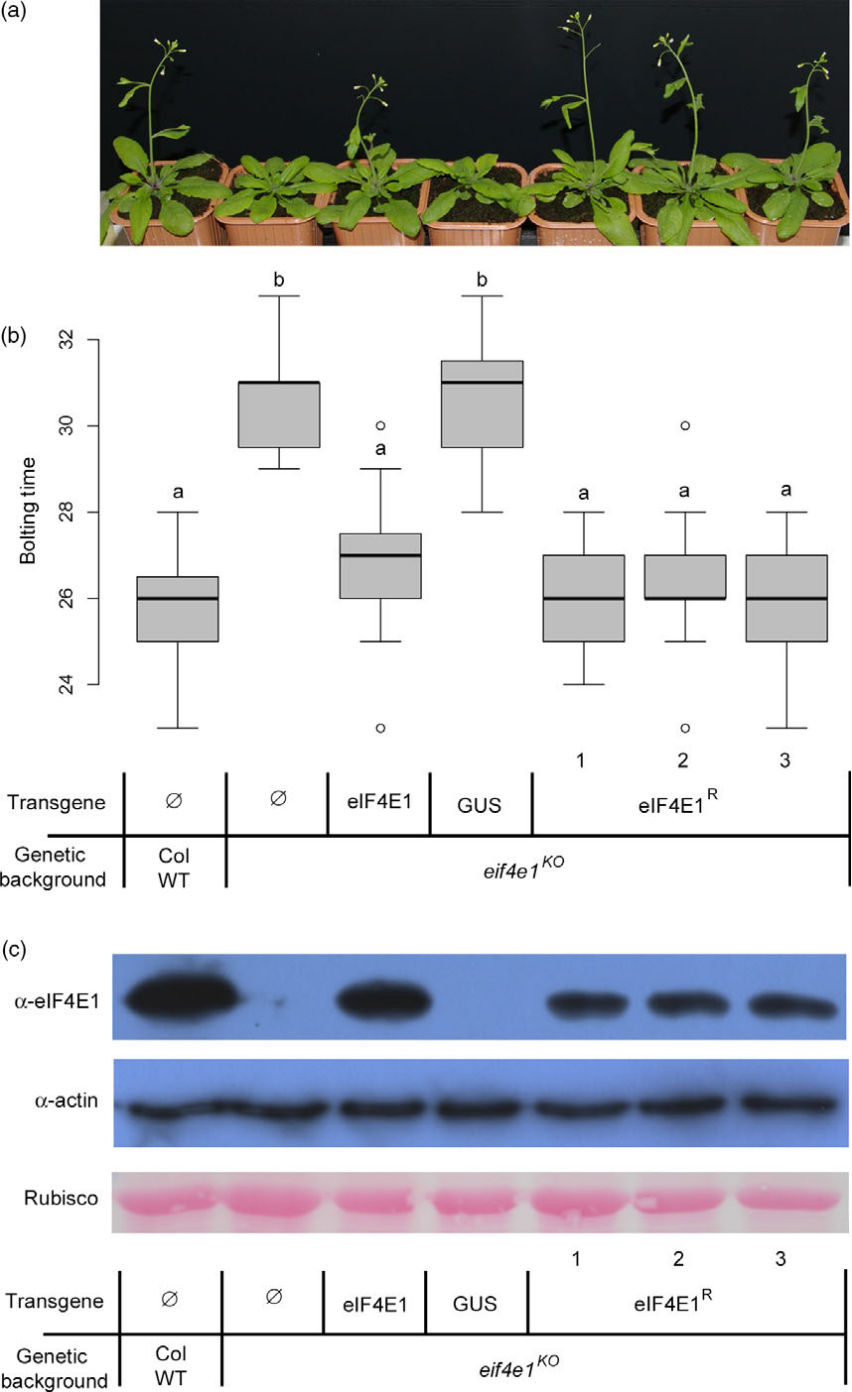
Functional complementation of *eif4e1* ^*KO*^ plants by *eIF4E1* ^*R*^ allele. (a) Bolting of *eif4e1* ^*KO*^ and complemented plants, 4 weeks after sowing. Columbia Wild‐type plants (Col WT) are used as control. Results are shown for three independent transgenic lines (1 to 3) expressing the eIF4E1^R^ construct in a *eif4e1* ^*KO*^ background. Table legend shown under (b) applies also for (a). (b) Boxplot representation of the bolting time (in days after sowing) for the same genotypes as in (a). Results are averaged from 16 individual plants per genotype. (a) and (b) represent significantly different groups (*P* < 0.05). (c) *In planta* cap‐binding purification of eIF4E1 proteins. Total soluble protein extract from control and transgenic plants was purified on m7GTP‐agarose beads. After purification, the output fraction was analysed by Western blot using anti‐eIF4E1 antibody while equal loading control was checked on total protein (input) by Western blot for actin detection and by Ponceau staining for Rubisco protein detection.

Although 4E translation initiation factors are largely redundant in their functions (Browning and Bailey‐Serres, 2015; Combe *et al*., 2005; Duprat *et al*., 2002; Nicaise *et al*., 2007), the *eif4e1* ^*KO*^ *Arabidopsis* plants were found to display a consistent 7‐day bolting delay when compared with the wild‐type Columbia accession (Col WT) (Figure 2a,b, Bastet *et al*., 2017). To assess whether *eIF4E1* ^*R*^ encodes a functional translation initiation factor, the ability of *eIF4E1* ^*R*^ to complement this bolting delay was tested. Indeed, *eIF4E1* ^*R*^ restored the wild‐type timing of bolting (Figure 2a,b). Furthermore, total protein extracts were subjected to cap analog‐affinity assay and, on all independent transgenic lines expressing either *eIF4E1* or *eIF4E1* ^*R*^, eIF4E1 proteins were shown to bind the cap analog, confirming that *eIF4E1* ^*R*^ encodes a functional protein (Figure 2c).

### *eIF4E1* ^*R*^ is a resistance allele to ClYVV

**Figure 3:**
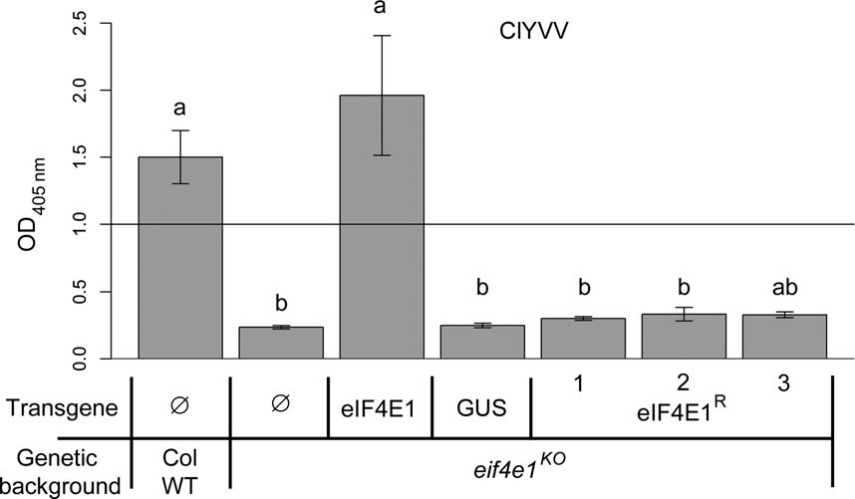
*eIF4E1* ^*R*^ is a resistance allele to ClYVV in *Arabidopsis*. ClYVV accumulation in *eif4e1* ^*KO*^ plants transformed with the wild‐type *eIF4E1* genomic construct under its own promoter (eIF4E1), the GUS gene under the control of the 35S promoter (GUS) or with the genomic *eIF4E1* ^*R*^ construct under its endogenous promoter (three independent lines are shown, eIF4E1^R^ 1–3). Wild‐type Columbia and *eif4e1* ^*KO*^ plants are used as positive and negative controls, respectively. Accumulation of ClYVV was assessed by DAS‐ELISA 30 days postinoculation. (a) and (b) represent significantly different groups, *P* < 0.05.

We next addressed whether the six mutations introduced in the synthetic allele were sufficient to confer resistance to ClYVV, a potyvirus that requires an eIF4E1 factor for its viral cycle. All plants were mechanically inoculated with ClYVV and the viral accumulation was measured 30 days postinoculation (dpi) by double antibody sandwich enzyme‐linked immunosorbent assay (DAS‐ELISA) (Figure 3). As expected, ClYVV accumulated in both Col WT and *eIF4E1*‐complemented plants while no virus accumulation was observed in *eif4e1* ^*KO*^ plants. Interestingly, no virus accumulation was detected in the *eif4e1* ^*KO*^ plants complemented by the *eIF4E1* ^*R*^ construct suggesting that this mutated factor cannot be recruited by ClYVV. Altogether, these results show that the six AA changes introduced in *eIF4E1* ^*R*^ are sufficient to convert eIF4E1 into a resistance allele to ClYVV, while maintaining its physiological functionality. This is similar to the resistance genes that have been previously identified in crops or in their wild relatives (Robaglia and Caranta, 2006).

### *eIF4E1* ^*R*^ rescues the lethality associated with the double mutation *eif4e1* ^*KO*^ *eifiso4e* ^*KO*^ and does not impact plant yield

**Figure 4:**
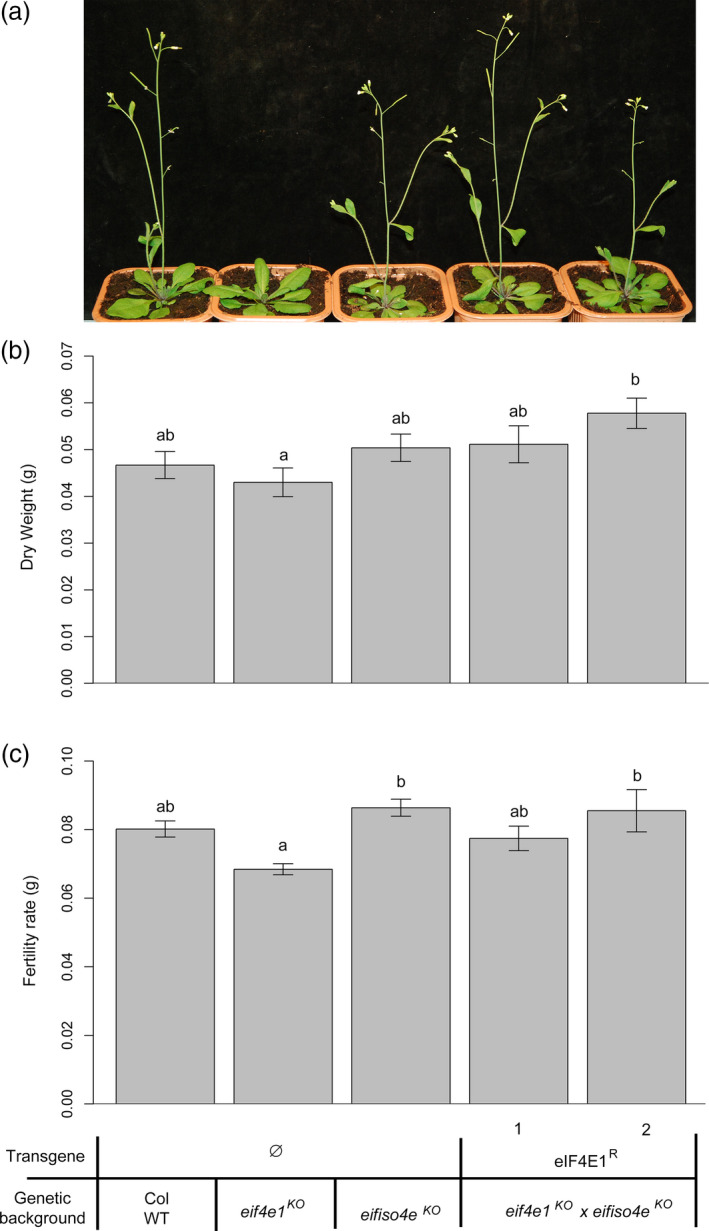
*eIF4E1* ^*R*^ complements the *eif4e1* ^*KO*^ *eifiso4e* ^*KO*^ lethality phenotype at no developmental cost. (a) Phenotype of two independent *eif4e1* ^*KO*^ *eifiso4e* ^*KO*^ *eIF4E1* ^*R*^ lines, four weeks after sowing. (b) Dry weight analyses of 4‐week‐old controls and *eif4e1* ^*KO*^ *eifiso4e* ^*KO*^ *eIF4E1* ^*R*^ plants. Results were averaged from 20 plants for each genotype. (c) Fertility rate is the weight of seeds produced by plants of controls lines and *eif4e1* ^*KO*^ *eifiso4e* ^*KO*^ *eIF4E1* ^*R*^ plants. Results were averaged from 10 plants for each genotype. Kruskal–Wallis statistical tests were performed to identify statistically significant differences (a) and (b) represent significantly different groups (*P* < 0.05, standard error bars are represented on the graph).

It was previously shown that the resistance spectra associated with either *eif4e1* ^*KO*^ or *eifiso4e* ^*KO*^, to ClYVV and *Turnip mosaic virus* (TuMV), respectively (Duprat *et al*., 2002; Lellis *et al*., 2002), could not be combined in the same plant because of plant lethality (Callot and Gallois, 2014; Patrick *et al*., 2014). We speculated that since *eIF4E1* ^*R*^ encodes a functional cap‐binding factor, it may successfully be combined with loss‐of‐function alleles affecting both *eIF4E1* and *eIFiso4E* and could therefore rescue the lethal phenotype. To verify this hypothesis, two previously described independent T2 transgenic lines [*eif4e1* ^*KO*^/*eif4e1* ^*KO*^ *eIF4E1* ^*R*^] (homozygous for *eif4e1* ^*KO*^ allele but complemented by *eIF4E1* ^*R*^) were crossed with [*eif4e1* ^*KO*^/*eif4e1* ^*KO*^ *eIFiso4E*/*eifiso4e* ^*KO*^] (homozygous for *eif4e1* ^*KO*^ but heterozygous for *eifiso4e* ^*KO*^) plants. The resulting F3 plants knocked out for *eIF4E1* and *eIFiso4E* but complemented by *eIF4E1* ^*R*^ were viable (Figures 4a and S3). The rescue of the lethal phenotype by *eIF4E1* ^*R*^ further confirmed that this allele was fully functional *in planta*. Consistently, the *eif4e1* ^*KO*^/*eif4e1* ^*KO*^ *eifiso4e* ^*KO*^/*eifiso4e* ^*KO*^ plants complemented by *eIF4E1* ^*R*^ (named hereafter *eif4e1* ^*KO*^ *eifiso4e* ^*KO*^ *eIF4E1* ^*R*^) did not show any delayed bolting phenotype compared with wild‐type plants (Figures 4a and S4). In addition, no decrease in dry weight or in seed yield was observed (Figure 4b,c and S4). In conclusion, the synthetic *eIF4E1* ^*R*^ allele can fully complement the lack of both *eIF4E1* and *eIFiso4E* isoforms *in planta*.

### Cumulative resistance to several potyviruses in *eIF4E1* ^*R*^‐complemented plants

**Figure 5:**
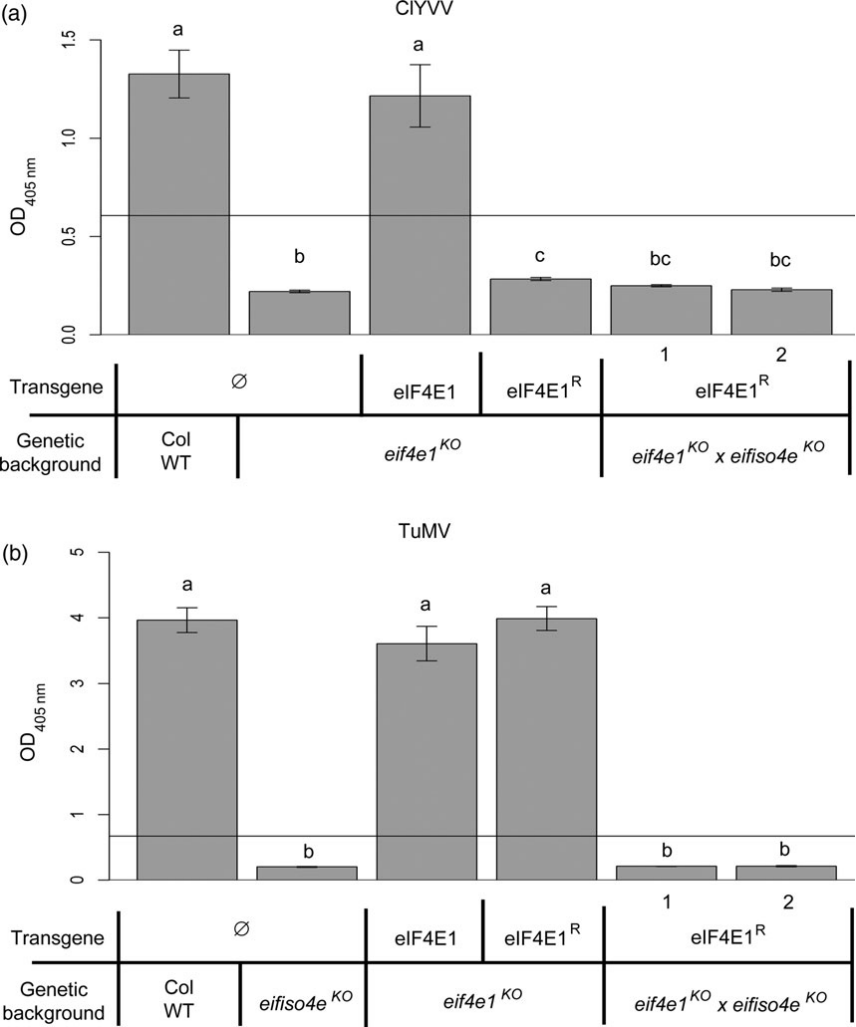
*eif4e1* ^*KO*^ *eifiso4e* ^*KO*^ *eIF4E1* ^*R*^ plants cumulate the respective resistances to TuMV and ClYVV. ClYVV and TuMV viral accumulation measured by DAS‐ELISA in control lines and in two *eif4e1* ^*KO*^ *eifiso4e* ^*KO*^ *eIF4E1* ^*R*^ lines. DAS‐ELISA was performed 30 days after inoculation with ClYVV (a) or 21 days after inoculation with TuMV (b). a, b and c represent significantly different groups, *P* < 0.05.

As eIF4E1 and eIFiso4E are required for ClYVV and TuMV multiplication, respectively, *eif4e1* ^*KO*^ *eifiso4e* ^*KO*^ *eIF4E1* ^*R*^ plants, together with control lines including single mutants affecting *eIF4E1* or *eIFiso4E* alone, were separately challenged with these two viruses, and the accumulation of viruses was measured by ELISA (Figure 5). As expected, Col WT plants were susceptible to both viruses while *eif4e1* ^*KO*^ and *eifiso4e* ^*KO*^ plants were resistant to ClYVV and TuMV, respectively. Remarkably, ClYVV or TuMV did not accumulate in the *eif4e1* ^*KO*^ *eifiso4e* ^*KO*^ *eIF4E1* ^*R*^ lines. *Lettuce mosaic virus* (LMV) and *Plum pox virus* (PPV) that both rely on the isoform eIFiso4E (Duprat *et al*., 2002; Nicaise *et al*., 2003) were further inoculated onto the similar mutated lines, and the results showed that the *eif4e1* ^*KO*^ *eifiso4e* ^*KO*^ *eIF4E1* ^*R*^ lines were fully resistant to these potyviruses as well (Figure S5). Therefore, by complementing the loss‐of‐function for both *eIF4E1* and *eIFiso4E*, the synthetic *eIF4E1* ^*R*^ allele allows combination of the resistance spectrum associated with each individual mutation, leading to an overall broad resistance spectrum to several potyviruses.

### *eIF4E1* ^*R*^ confers resistance to a potyvirus relying on both *eIF4E1* and *eIFiso4E*

**Figure 6:**
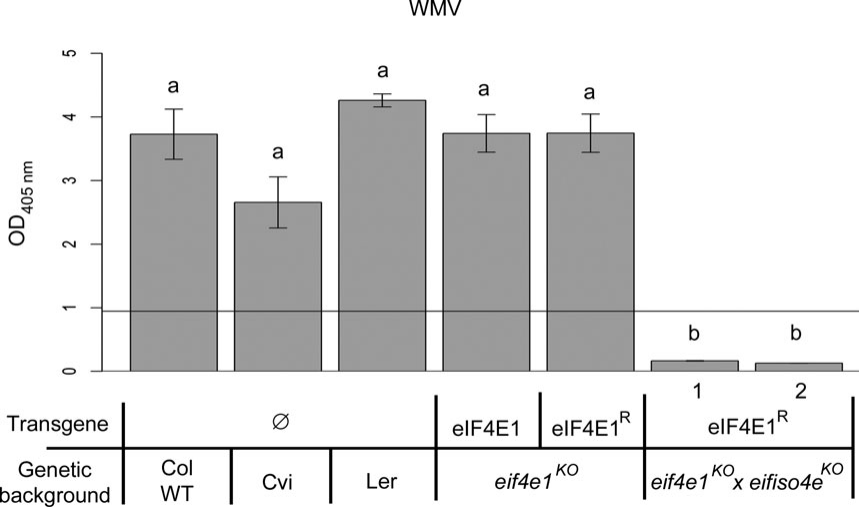
*eif4e1* ^*KO*^ *eifiso4e* ^*KO*^ *eIF4E1* ^*R*^ plants resistance spectrum extends to WMV. WMV viral accumulation was measured by DAS‐ELISA in control lines and in two *eif4e1* ^*KO*^ *eifiso4e* ^*KO*^ *eIF4E1* ^*R*^ lines. DAS‐ELISA was performed at 21 days after inoculation with WMV. a and b represent significantly different groups, *P* < 0.05.

*Watermelon mosaic virus* (WMV) is a potyvirus that mainly affects cucurbit plants, but also *Arabidopsis*. Previously, the *Arabidopsis* Cap Verde island accession (Cvi) was identified as a source of partial resistance to WMV relying on the *chloroplastic PHOSPHOGLYCERATE KINASE 2* gene (*cPGK2*) (Ouibrahim *et al*., 2014). Unlike most potyviruses, no resistance to WMV associated with loss‐of‐function alleles of either *eIF4E1* or *eIFiso4E* has been shown so far (Figure S6). A hypothesis is that WMV could recruit either eIF4E1 or eIFiso4E as do *Pepper veinal mottle virus* (PepVMV) or *Chilli veinal mottle virus* (ChiVMV) in *Capsicum* species (Hwang *et al*., 2009; Ruffel *et al*., 2006). In agreement with this assumption, the *eif4e1* ^*KO*^ *eifiso4e* ^*KO*^ *eIF4E1* ^*R*^ lines were also fully resistant to WMV since no virus accumulated in the inoculated plants 3 weeks postinoculation (Figure 6). This result shows that, beyond allowing the pyramiding of the resistance spectra conferred by both mutated eIF4E and eIFiso4E, the strategy outlined here can extend the resistance to potyviruses using both factors.

### *eIF4E1* ^*R*^ restrains TuMV resistance‐breaking isolates

The durability of resistance is a major issue for plant breeding and it is crucial to develop and release resistances that are efficient towards resistance‐breaking (RB) emerging isolates. Significantly, eIF4E‐mediated resistance can be overcome by RB potyviruses (Sanfaçon, 2015; Wang and Krishnaswamy, 2012). Previously, two RB isolates of TuMV able to overcome *eifiso4e* ^*KO*^‐mediated resistance were isolated in *Arabidopsis* (Gallois *et al*., 2010). Each isolate, TuMV‐E116Q and TuMV‐N163Y, possesses a single nonsynonymous mutation (mutation E116Q or N163Y, respectively) in the avirulence factor VPg (viral genome‐linked protein), that is responsible for the resistance overcoming. It had been hypothesized that the overcoming of *eifiso4e* ^*KO*^‐mediated resistance could be achieved by the ability of both isolates to recruit another translation initiation factor, namely eIF4E1 (Gallois *et al*., 2010).

**Figure 7:**
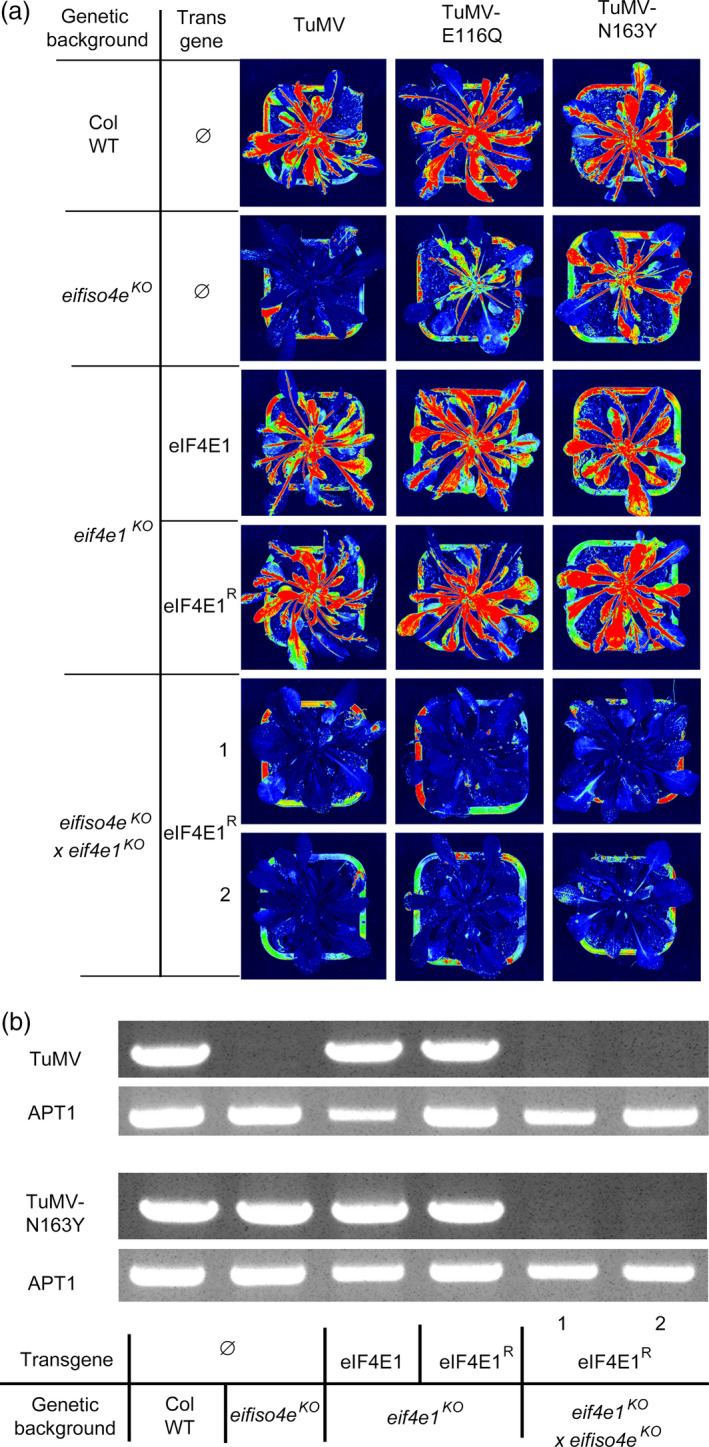
*eif4e1* ^*KO*^ *eifiso4e* ^*KO*^ *eIF4E1* ^*R*^ plant resistance spectrum extends to resistance‐breaking TuMV isolates. Controls and *eif4e1* ^*KO*^ *eifiso4e* ^*KO*^ *eIF4E1* ^*R*^ lines were challenged with either GFP‐tagged TuMV, GFP‐TuMV‐E116Q (RB) or GFP‐TuMV‐N163Y (RB). Viral accumulation was assessed using GFPcam (PSI) camera‐imaging (a). Fluorescence intensity is shown in false colour from blue (low) to red (high intensity) with the plant pot reflecting light, allowing visualization of the outline of the plant position. (b) Detection of TuMV mRNA by RT‐PCR amplification of the VPg coding sequence for both TuMV‐GFP and TuMV‐N163Y‐GFP 21 dpi in the noninoculated leaves. *ADENINE PHOSPHORIBOSYL TRANSFERASE 1 (APT1)* gene was used as a control for RNA extraction.

As often with resistance‐breaking strains (Moury *et al*., 2004), TuMV‐E116Q and TuMV‐N163Y accumulate weakly in *eifiso4e* ^*KO*^ lines (Figure S7). Therefore, to monitor plant infection, we inoculated the same plants as before with a viral isolate expressing the GFP reporter gene. The mutations E116Q and N163Y were each introduced into a TuMV‐GFP infectious cDNA clone (Beauchemin *et al*., 2005), generating TuMV‐E116Q‐GFP and TuMV‐N163Y‐GFP, respectively. Plants were agro‐inoculated, and the GFP accumulation in plants was detected 21 dpi using a GFP‐imaging camera (Figure 7a). TuMV‐E116Q‐GFP and TuMV‐N163Y‐GFP, but not TuMV‐GFP accumulated in *eifiso4e* ^*KO*^ plants, confirming their ability to overcome the *eifiso4e* ^*KO*^‐mediated resistance. However, the two *eif4e1* ^*KO*^ *eifiso4e* ^*KO*^ *eIF4E1* ^*R*^ lines were fully resistant to both resistance‐breaking TuMV isolates. The absence of virus accumulation in the *eif4e1* ^*KO*^ *eifiso4e* ^*KO*^ *eIF4E1* ^*R*^ lines was moreover confirmed by RT‐PCR on total RNA extracted from the inoculated plants (Figure 7b). Altogether, our results show that one nonsynonymous mutation either at position 116 or 163 in the VPg allows RB‐TuMV viruses to use a new translation initiation factor, eIF4E1, in addition to eIFiso4E, and that the engineered *eif4e1* ^*KO*^ *eifiso4e* ^*KO*^ *eIF4E1* ^*R*^ genotype could significantly improve virus resistance durability.

### *eIF4E1* ^*R*^ confers resistance to one polerovirus species

Translation initiation factors 4E are mostly associated with resistance to potyviruses but resistances to other ssRNA+ viruses, harbouring different genomic features, have also been described (Robaglia and Caranta, 2006). For example, loss‐of‐function in *eIF4E1* was shown to be implicated in a delay of susceptibility to the carmovirus *Turnip crinkle virus* (TCV) (Yoshii *et al*., 1998). We found that the engineered *eif4e1* ^*KO*^ *eifiso4e* ^*KO*^ *eIF4E1* ^*R*^ plants were fully susceptible to TCV, showing that the *eIF4E1* ^*R*^ allele does not constitute a resistance allele to TCV (Figure S8). Likewise, the engineered *eif4e1* ^*KO*^ *eifiso4e* ^*KO*^ *eIF4E1* ^*R*^ plants were fully susceptible to *Grapevine fanleaf virus* (GFLV) and *Arabis mosaic virus* (ArMV), two ssRNA+ nepoviruses (Figure S9).

Finally, the role of translation initiation factors in resistance to polerovirus, such as *Turnip yellows virus* (TuYV), *Beet mild yellowing virus* (BMYV) and *Beet western yellows virus*‐USA (BWYV‐USA), had previously been investigated in *Arabidopsis* (Reinbold *et al*., 2013). TuYV was shown to require the *Arabidopsis* eIFiso4G1 to infect the plant, whereas BMYV and BWYV infection was compromised in plants devoid of functional eIF4E1, although the latter resistance was only partial. To assess whether *eIF4E1* ^*R*^ could confer resistance to viruses other than potyviruses, the *eif4e1* ^*KO*^ *eifiso4e* ^*KO*^ *eIF4E1* ^*R*^ lines were challenged with these three polerovirus species.

**Figure 8:**
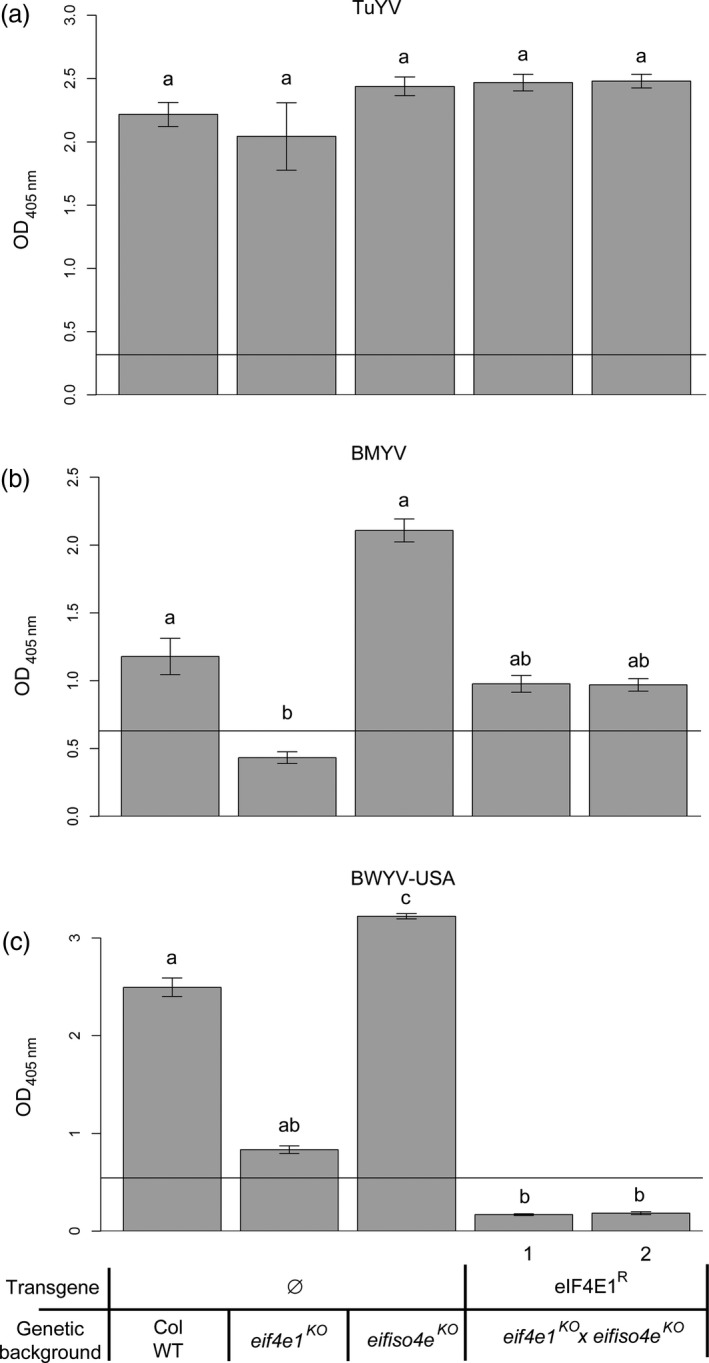
*eif4e1* ^*KO*^ *eifiso4e* ^*KO*^ *eIF4E1* ^*R*^ plants resistance spectrum extends to polerovirus species BWYV‐USA but not to TuYV and BMYV. BWYV‐USA, TuYV and BMYV viral accumulation was measured by DAS‐ELISA in control lines and in two *eif4e1* ^*KO*^ *eifiso4e* ^*KO*^ *eIF4E1* ^*R*^ lines. DAS‐ELISA was performed 21 days after inoculation with TuYV (a), BMYV (b) or BWYV‐USA (c). 12 plants per genotype were tested, and experiments were repeated twice. a, b and c represent significantly different groups, *P* < 0.05.

Aphid‐mediated inoculation was performed on controls and *eif4e1* ^*KO*^ *eifiso4e* ^*KO*^ *eIF4E1* ^*R*^ plants. Viral accumulation was detected by DAS‐ELISA 21 dpi (Figure 8). All genotypes were fully susceptible to TuYV, consistent with the virus requirement for eIFiso4G1 and not eIF4E1 or eIFiso4E. Intermediate resistance to BMYV and BWYV‐USA associated with *eIF4E1* loss‐of‐function was confirmed as described before (Reinbold *et al*., 2013) but interestingly while susceptibility to BMYV was restored in *eif4e1* ^*KO*^ *eifiso4e* ^*KO*^ *eIF4E1* ^*R*^ lines, this genotype was fully resistant to BWYV‐USA. In conclusion, the presence of the synthetic resistance allele *eIF4E1* ^*R*^ in an *eif4e1* ^*KO*^ *eifiso4e* ^*KO*^ genetic background is associated with a full resistance to a polerovirus isolate.

## Discussion

Natural eIF4E‐based resistance alleles are largely used to introduce virus resistance in crops by breeding. Genetic engineering could help provide new sources of resistance in crops that are devoid of such natural resistance. In this study, we developed a synthetic allele, *eIF4E1* ^*R*^ that can make up for the lack of natural resistance alleles, and we gave evidence of its effectiveness in an *Arabidopsis*‐based pathosystem. We showed that *eIF4E1* ^*R*^ mimics natural resistance alleles when replacing endogenous eIF4E while maintaining biochemical (binding to cap analog) and physiological (seed yield, biomass) functions. A full assessment of this genotype's resistance to biotic or abiotic stresses as well as of any changes in translatome could allow to analyse more subtle changes in the plant physiology that could be associated with this new allele.

**Figure 9:**
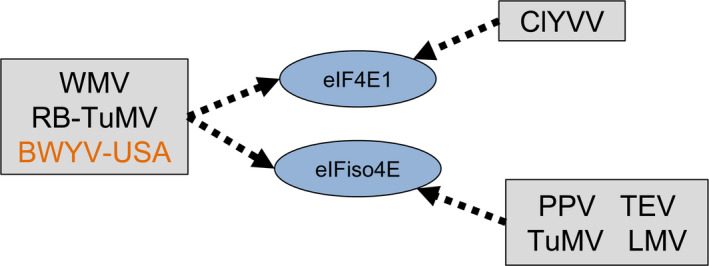
Update on the selective recruitment of translation initiation factors 4E by ssRNA+ viruses in *Arabidopsis*. Arrows indicate that the virus isolate relies on the 4E factor to infect the plant. WMV,* Watermelon mosaic virus*; RB‐TuMV, resistance‐breaking *Turnip mosaic virus;* ClYVV,* Clover yellow vein virus;* PPV,* Plum pox virus*; TEV,* Tobacco etch virus;* LMV,* Lettuce mosaic virus;* BWYV‐USA,* Beet western yellows virus*‐USA. Potyviruses are coloured in black and poleroviruses in orange.

By being fully functional, *eIF4E1* ^*R*^ allows combination of the resistance spectra associated with both loss‐of‐function alleles in *eIF4E1* and *eIFiso4E* (*i.e*. the resistance to ClYVV associated with *eif4e1* ^*KO*^ and the resistance to TuMV, PPV and LMV associated with *eifiso4e* ^*KO*^). Such association had been infeasible so far because of the lethality of the double mutant (Callot and Gallois, 2014; Patrick *et al*., 2014). Moreover, the same genetic combination involving *eIF4E1* ^*R*^ allowed us to extend the *Arabidopsis* resistance to WMV, a potyvirus for which no 4E‐mediated resistance has been identified so far. Interestingly, this resistance is more efficient than the one previously described based on the plant host factor *chloroplastic PHOSPHOGLYCERATE KINASE 2* (Ouibrahim *et al*., 2014). This shows that WMV is able to use the two main translation initiation factors in *Arabidopsis* indifferently, namely eIF4E1 and eIFiso4E isoforms. This is a very similar situation to the one that has been described for susceptibility to PepVMV and ChiVMV in pepper, where resistance relies on both a *Ca‐eIF4E1‐pvr2* functional resistance allele and a natural *Ca‐eIFiso4E*‐*pvr6* KO allele (Hwang *et al*., 2009; Ruffel *et al*., 2006). We therefore suspect that the ability of certain viral species to recruit several isoforms could be more frequent than expected, and by generalization, the genericity of 4E factors in susceptibility to ssRNA+ viruses could be more universal than previously thought (Figure 9).

Finally, we described earlier that TuMV can overcome *eIFiso4E* loss‐of‐function through either one of two nonsynonymous mutations in the viral VPg coding sequence (Gallois *et al*., 2010). We hypothesized that because eIFiso4E was no longer available, the viral mutations associated with resistance breaking may extend the VPg's ability to recruit new host factors, possibly other translation initiation factors. Indeed, we show here that *eif4e1* ^*KO*^ *eifiso4e* ^*KO*^ *eIF4E1* ^*R*^ lines were fully resistant to both RB‐TuMV isolates. This means that E116Q and N163Y mutations in the VPg are two independent paths to the same RB mechanism, allowing the virus to recruit eIF4E1 as well as eIFiso4E in the plant. eIF4E‐based resistance breaking has often been associated with the recruitment of the same eIF4E factor, as shown by the pepper/potyvirus co‐evolution process (Charron *et al*., 2008). This work presents for the first time RB events associated with the recruitment of another 4E isoform. This allows us to anticipate that resistance based on eIF4E loss‐of‐function could be easily overcome, leading to serious consequences for the design of durable genetic resistance (Bastet *et al*., 2017). In conclusion, the genetic combination using the synthetic allele designed in this study is associated with a full resistance to all seven potyviruses tested. Overall, our results show that combining mutations in several 4E translation initiation factors is highly likely to expand the resistance spectrum to other potyviruses, but might also extend the resistance durability.

More generally, translation initiation factors were also found in crops and in *Arabidopsis* to be involved in resistance to ssRNA+ viruses with different genome organizations to potyviruses (Robaglia and Caranta, 2006). This includes the resistance to viruses from genera such as *Polerovirus* (family *Luteoviridae*) and *Sobemovirus* (family *Tombusviridae*), harbouring a much shorter VPg (around 90 AA in length) displaying little homology with the *Potyvirus* VPg (around 190 AA). This also extends to *Carmovirus* (family *Tombusviridae*) members, including *Melon necrotic mosaic virus,* whose genomes are uncapped (Jiang and Laliberté, 2011). To demonstrate whether the efficiency of the *eIF4E1* ^*R*^ allele, specifically designed as a resistance allele to potyviruses, could be extended to other ssRNA+ viruses, we challenged the *eIF4E1* ^*R*^‐complemented plants with polerovirus species (TuYV, BMYV, BWYV‐USA), nepoviruses (GFLV‐F13, GFLV‐GHu, ArMV) and carmovirus (TCV). We did not observe any resistance to TCV, nor to the three nepoviruses assayed. However, we found a complete resistance to one polerovirus species (BWYV‐USA) out of the three tested. It is worth noting that the same polerovirus species have been previously used to challenge loss‐of‐function mutants affecting translation initiation factors in *Arabidopsis*, with the *eif4e1* ^*KO*^ mutant showing partial resistance to BMYV and BWYV‐USA (Reinbold *et al*., 2013). Significantly, in our study, *eIF4E1* ^*R*^ complementation restores full susceptibility to BMYV while it triggers full resistance to BWYV‐USA, which could be of interest for sugarbeet breeding programmes. Recently, potyvirus VPg has been suggested to interact with host eIF4E through a conserved noncanonical 4E‐binding domain (Miras *et al*., 2017) which interestingly does not seem to be conserved in VPg from other groups of ssRNA+ viruses. Other viruses are likely to recruit eIF4E through different structural binding domains and would therefore only be marginally affected by potyvirus‐specific nonsynonymous AA changes in eIF4E regions I and II as the ones introduced in the synthetic *eIF4E1* ^*R*^ allele (Robaglia and Caranta, 2006). For example, the *Arabidopsis* eIFiso4E binds the viral protease rather than the VPg of the nepovirus *Tomato ringspot virus* (Léonard *et al*., 2002) and the carmovirus *Melon necrotic spot virus* (MNSV) binds eIF4E directly through an RNA motif at the 3′ end of the viral genome (Truniger *et al*., 2008). Consistent with this, the melon *eIF4E* natural allele *nsv* associated with resistance to MNSV displays a very idiosyncratic AA change at the C‐terminal end of the eIF4E protein sequence (Nieto *et al*., 2006). This suggests that, to adjust to the different interaction patterns developed by these viruses, specific additional mutations—possibly outside regions I and II—should be characterized and introduced into eIF4E or eIFiso4E protein sequences, to tailor specific resistances. Besides, ssRNA+ viruses can also use other translation initiation factors. For example, sobemovirus *Rice yellow mottle virus* in rice and polerovirus TuYV in *Arabidopsis* both use the large scaffolding translation initiation factors eIFiso4G through the binding of their VPgs (Hébrard *et al*., 2010; Reinbold *et al*., 2013), suggesting that these factors may also be designed to trigger specific resistance.

The innovative approaches developed here can be fully translated to crops in order to develop amenable resistances to control losses associated with virus infection using environment friendly strategies. Mostly, by targeting simultaneously several translation initiation factors 4E and by combining loss‐of‐function alleles with modified functional alleles, our approach is likely to trigger resistance without impairing crop agronomical performance, a major requisite for plant breeding. By introducing six independent mutations in the eIF4E1 coding sequence, similar to those naturally present in pea, the *eIF4E1* ^*R*^ synthetic allele confers a very robust resistance to potyviruses. We show that mutations associated with resistance can be easily transferred from one species to another to design alleles based on the endogenous relevant genes, as previously shown in *Solanaceae* (Cavatorta *et al*., 2011; Kang *et al*., 2007). Furthermore, by expressing the modified eIF4E1^R^ under the control of its own regulatory sequences, the complementation of a knockout allele can be easily performed without the use of foreign DNA sequences such as strong constitutive promoters and terminators. This approach, also called cis‐genesis, constitutes a major issue for public acceptance (Ilardi and Tavazza, 2015; Schouten *et al*., 2006) and a strategy similar to the one developed in this study in *Arabidopsis* could be easily transposed to any crops. This can also be carried out by new breeding techniques (NBT) such as CRISPR/Cas9 technology particularly with the use of Cas9‐cytidine deaminase fusion, allowing precise base substitution *in situ* in the genome of plants of agronomical interest (Komor *et al*., 2016; Murovec *et al*., 2017; Shimatani *et al*., 2017). Genetic diversity could then be optimally exploited in a trans‐species manner to provide new engineered sources of resistance.

## Experimental procedure

### Plant materials and growth conditions

*Arabidopsis thaliana* Columbia‐0 (Col) plants were used in this study as a wild‐type control for all tests. *A. thaliana* accessions Landsberg *erecta* (L*er*) and Cap Verde island (Cvi) were also used as susceptible and resistant controls, respectively, for WMV infection according to (Ouibrahim *et al*., 2014). *eif4e1* ^*KO*^ and *eifiso4e* ^*KO*^ lines are, respectively, homozygous for the T‐DNA insertion in *eIF4E1* (At1g18040; SALK_145583) and the transposon dSpm insertion in *eIFiso4E* (At5g35620; Duprat *et al*., 2002) in a Col background. For genetic crosses, immature flowers were emasculated and cross‐pollinated manually.

For growth on plates, seeds were surface‐sterilized for 10 min in 95% ethanol with 0.1% Tween 20 and plated on Murashige and Skoog (MS) medium, supplemented if necessary with 10 mg/L hygromycin B. Seedlings were transferred to soil after 2 weeks. For nepovirus, polerovirus, TCV, PPV and LMV resistance assays, seeds were directly sowed on soil and individually transferred 2 weeks later.

Plants were grown in a culture chamber at 20–24 °C with a cycle of 16‐h light (fluorescent light at 100 μmol photon/m^2^/s) and 8‐h dark for the flowering assay and nepovirus resistance assay, with a cycle of 8‐h light and 16‐h dark for ClYVV, TuMV, WMV, RB‐TuMV, LMV, PPV, and TCV and 10‐h light and 14‐h dark for TuYV, BMYV‐2itb and BWYV‐USA resistance assays. For the flowering and fresh/dry weight assays, the different genotypes of plants were randomized in the culture chamber.

### Alignment data and 3D protein modelling

The sequences for pea eIF4E from resistant and susceptible lines were collected from Gao *et al*. (2004) (GenBank ID AY611423, AY423375 and AY611425). Protein alignments were carried out using MultiAlin (http://multalin.toulouse.inra.fr) and BoxShade (http://www.ch.embnet.org/software/BOX_form.html).

The 3D modelization of AteIF4E1 and eIF4E1^R^ protein was constructed by homology prediction with YASARA software (http://www.yasara.org; Krieger and Vriend, 2014) using the crystal structure of *Pisum sativum* eIF4E1 (PDB ID: 2WMC‐C) as a template. Protein structure was visualized with PyMOL software (The PyMOL Molecular Graphics System, version 1.8, Schrodinger, 2015; https://www.pymol.org/; Schrodinger, 2015).

### Plasmid construction and plant transformation

A 3533‐bp genomic –At4g18040‐ *eIF4E1* fragment (spanning 1500 bp of the promoter region and 150 bp of the 3′UTR) was amplified on Columbia genomic DNA using primers Z4148‐F and Z4148‐R (Table S1) and subcloned into pDONR207 using BP gateway recombination (Invitrogen), resulting in plasmid pJL631. Site‐directed mutagenesis associated with the following AA changes—W69L T80D S81D S84A G114R and N176K—were iteratively introduced with the QuikChange II Site‐Directed Mutagenesis Kit (Stratagene) using specific primers. All primers are listed in Table S1. All constructs were sequence‐checked. The resulting *eIF4E1* ^*R*^ genomic construct was then cloned into the binary vector pMDC099 (Curtis and Grossniklaus, 2003) using gateway LR recombination and subsequently introduced in *Arabidopsis* genome by Floral Dip agrotransformation (Clough and Bent, 1998). Transformants were selected on MS plates supplemented with 10 mg/L hygromycin B.

### Virus materials, inoculation and detection assays

ClYVV provided by Ichiro Uyeda (Sato *et al*., 2005), TuMV (CDN1 isolate) and WMV (Fr isolate) were propagated on *Nicotiana benthamiana* cv Xanthi, turnip (*Brassica rapa*) and zucchini squash (*Cucurbita pepo*), respectively, prior to being rubbed‐inoculated on leaves from 1‐month‐old *Arabidopsis* plants (Gallois *et al*., 2010; Ouibrahim *et al*., 2014). Viral accumulation detection of ClYVV, TuMV and WMV was carried out 20–30 days after inoculation by ELISA using anti‐ClYVV (DSMZ), anti‐potyvirus group (Agdia, Elkhart, Indiana) and anti‐WMV (SEDIAG, Longvic, France, http://www.sediag.com) antisera and detection sets, respectively.

The LMV‐AFVAR1 isolate, differing by a single amino acid change in the CP N‐terminal region from its LMV‐AF199 progenitor (Decroocq *et al*., 2009), was selected for its capacity to systemically infect Columbia‐0. This virus was inoculated mechanically onto *Arabidopsis* rosette leaves 6 weeks after sowing. Virus accumulation in apical noninoculated leaves was analysed by DAS‐ELISA assay with polyclonal antibodies anti‐LMV (German‐Retana *et al*., 2008) 21 days postinoculation (dpi).

The PPV‐R isolate (Dideron strain) was agro‐inoculated using the pBINPPVnkGFP construct (Fernández‐Fernández *et al*., 2001) and ELISA assays were performed 21 dpi, for apical noninoculated leaves, using commercial anti‐PPV antibodies (D+M polyclonal antibody, LCA Laboratory, Blanquefort, France).

RB‐TuMV plasmids were constructed from pCambia TuMV‐GFP, a gift from Jean‐François Laliberté (Beauchemin *et al*., 2005), by introducing into the VPg coding sequence the E116Q and N163Y mutations, respectively, by PCR‐based mutagenesis using the QuikChange^®^ II XL Site‐Directed Mutagenesis kit (Agilent, Santa Clara, CA) with primers Z4653‐F/Z4653‐R for E116Q and Z4654‐F/Z4654‐R for N163Y. Presence of the mutations was checked by sequencing (Genoscreen, Lille, France), and the resistance‐breaking properties of these variants were assessed on *eifiso4e* ^*KO*^ plants. The plasmids were transformed into the C58S pMP90 agrobacterium strain and agro‐inoculated in leaves. GFP accumulation in plants was imaged 3 weeks after inoculation using a closed fluorometric camera FluorCam FC 800‐C/1010‐GFP (Photon System Instruments, Drasov, Czech Republic) equipped with a GFP filter. Fluorescence is represented in false colours.

Polerovirus inoculations were carried out as previously described (Reinbold *et al*., 2013). Briefly, BMYV (isolate 2ITB), BWYV‐USA and TuYV were inoculated using *Myzus persicae* membrane‐fed on purified virus for 24 h. Ten viruliferous aphids were then transferred onto each test plant for an inoculation period of 4 days. Detection of viral infection was performed by DAS‐ELISA (Loewe, Kronach, Germany) 21 dpi.

For nepoviruses, plants were mechanically inoculated with crude saps from *Chenopodium quinoa* infected separately with GFLV‐F13, GFLV‐GHu and ArMV‐Tanat isolates. Detection of viral infection was performed by DAS‐ELISA using anti‐GFLV or anti‐ArMV polyclonal antibodies (Bioreba AG, Switzerland) 19 dpi for GFLV isolates and 24 dpi for ArMV.

TCV isolate M (Oh *et al*., 1995) inoculation with infectious clone contained in pBIN61 was carried out by agro‐infiltration on 5‐week‐old plants. Infection was detected by symptom development 23 dpi.

All results presented are mean values from at least 15 independent plants per genotype, unless indicated otherwise, and error bars represent standard errors. The threshold for susceptibility is represented by a line on each graph and refers to absorbance value at 405 nm in ELISA equal to three times the mean value for healthy controls.

### Expression analysis by Reverse transcription

Total RNA was extracted from young noninoculated leaves of 1‐month‐old plants using TRI‐Reagent (Sigma‐Aldrich, St Louis, MO). RT‐PCR was performed with AMV reverse transcriptase (Avian myeloblastosis virus, Promega) on 1 μg of RNA according to the supplier's instructions. *eIF4E1* (At4g18040) and, as a control, *ADENINE PHOSPHORYBOSYL TRANSFERASE 1* (*APT1*, At1g27450) cDNAs were amplified with primers Z3135‐F/Z3135‐R and Z1734/Z1735, respectively.

A 575‐bp fragment covering the central region of the TuMV VPg coding sequence was similarly amplified by RT‐PCR from inoculated plants with primers Z4735‐F/Z4735‐R.

### Antibodies and Western blot analysis

Total protein extracts in Laemmli buffer from 4‐week‐old plantlets were used for Western blot analysis. Electrophoresis by SDS‐PAGE was performed on equal amount of protein extracts, and proteins were transferred to Amersham™ Protran^®^ Premium nitrocellulose membranes (GE Healthcare, Chicago, Illinois, United States). To ensure the correct transfer of proteins and equal loading, membranes were stained with Ponceau S solution (Sigma‐Aldrich, St Louis, MO), before being incubated with anti‐actin or anti‐eIF4E1 antibodies at 1/5000 and 1/2000 dilution, respectively. Membranes were then incubated with goat horseradish peroxidase‐labelled anti‐mouse serum for detection of actin (1/5000 dilution) (Sigma‐Aldrich, St Louis, MO) and goat horseradish peroxidase‐labelled anti‐rabbit serum for detection of eIF4E1 (1/2000 dilution) (Sigma‐Aldrich, St Louis, MO). Horseradish peroxidase activity detection was carried out using a LumiGLO Reserve chemiluminescent substrate kit (KPL, www.kpl.com) and X–OMAT LS films (Kodak, New York).

The polyclonal serum targeted against the full *Arabidopsis thaliana eIF4E1* protein was produced by Proteogenix (Oberhausbergen, France) using the method previously described (Estevan *et al*., 2014).

### Cap‐binding assay

Total protein extracts obtained by grinding 4‐week‐old plants were suspended in a 40 mm HEPES/KOH pH 7.6, 100 mm KCl, 1 mm dithiothreitol, 10% glycerol, 1% phenylmethanesulphonyl fluoride and protease inhibitor cocktail (Roche, Basel, Switzerland) resuspension buffer. Samples were centrifuged for 10 min at 15000 g, and the supernatant fractions (corresponding to INPUT fraction) were added to 50 μL of 7 methyl‐GTP sepharose beads (GE Healthcare, Chicago, Illinois, United States) and incubated overnight at 4 °C. After four washes with the resuspension buffer detailed above, samples were centrifuged 1 min at 15 000 ***g*** at 4 °C. Pellets were resuspended in 30 μL of Laemmli buffer before being boiled for 5 min to elute proteins bound to the cap analog (corresponding to OUTPUT fraction). Protein detection was carried out by Western blot.

### Dry/fresh weight and fertility rate measurements

Three‐week‐old plants (20 per genotype) were cut, and aerial parts were weighed to calculate fresh weights. Each plant was then dried for 24 h in a 100 °C heating chamber before evaluating the dry weight.

Total seed production of 10 plants per genotype was assayed by collecting and weighing all the seeds of each plant. A pool of 100 seeds per genotype was also weighed to ensure that there was no difference in seed average weight between genotypes.

### Statistical analyses

Kruskal–Wallis statistical tests were performed using the pgirmess package on the free software R (http://www.r-project.org/).

## Supporting information


**Figure S1** Three‐dimensional predicted structure of *Pisum sativum* eIF4E proteins.

**Figure S2** *eIF4E1* expression analysis in controls and complemented lines.

**Figure S3** Genotyping of *eif4e1* ^*KO*^ *eifiso4e* ^*KO*^ *eIF4E1* ^*R*^ plants.

**Figure S4** Phenotype of the *eif4e1* ^*KO*^ *eifiso4e* ^*KO*^ *eIF4E1* ^*R*^ plants.

**Figure S5** *eif4e1* ^*KO*^ *eifiso4e* ^*KO*^ *eIF4E1* ^*R*^ plants resistance spectrum extends to LMV and PPV.

**Figure S6** The Cvi accession is partly resistant to WMV, but *eif4e1* ^*KO*^ and *eifiso4e* ^*KO*^ single mutants are both susceptible.

**Figure S7** Low accumulation of resistance‐breaking TuMV isolates in *eifiso4e* ^*KO*^ plants as detected by DAS‐ELISA

**Figure S8** *eif4e1* ^*KO*^ *eifiso4e* ^*KO*^ *eIF4E1* ^*R*^ plants resistance spectrum does not extend to carmovirus TCV.

**Figure S9** *eif4e1* ^*KO*^ *eifiso4e* ^*KO*^ *eIF4E1* ^*R*^ plants resistance spectrum does not extend to nepoviruses.

**Table S1** List of oligonucleotides used in this study.
