# Supplementary figures and images for: Trans‐species synthetic gene design allows resistance pyramiding and broad‐spectrum engineering of virus resistance in plants
Source: Plant Biotechnol J. 2018 Mar 5;16(9):1569–81. doi: 10.1111/pbi.12896 (PMC6097130; doi:10.1111/pbi.12896)

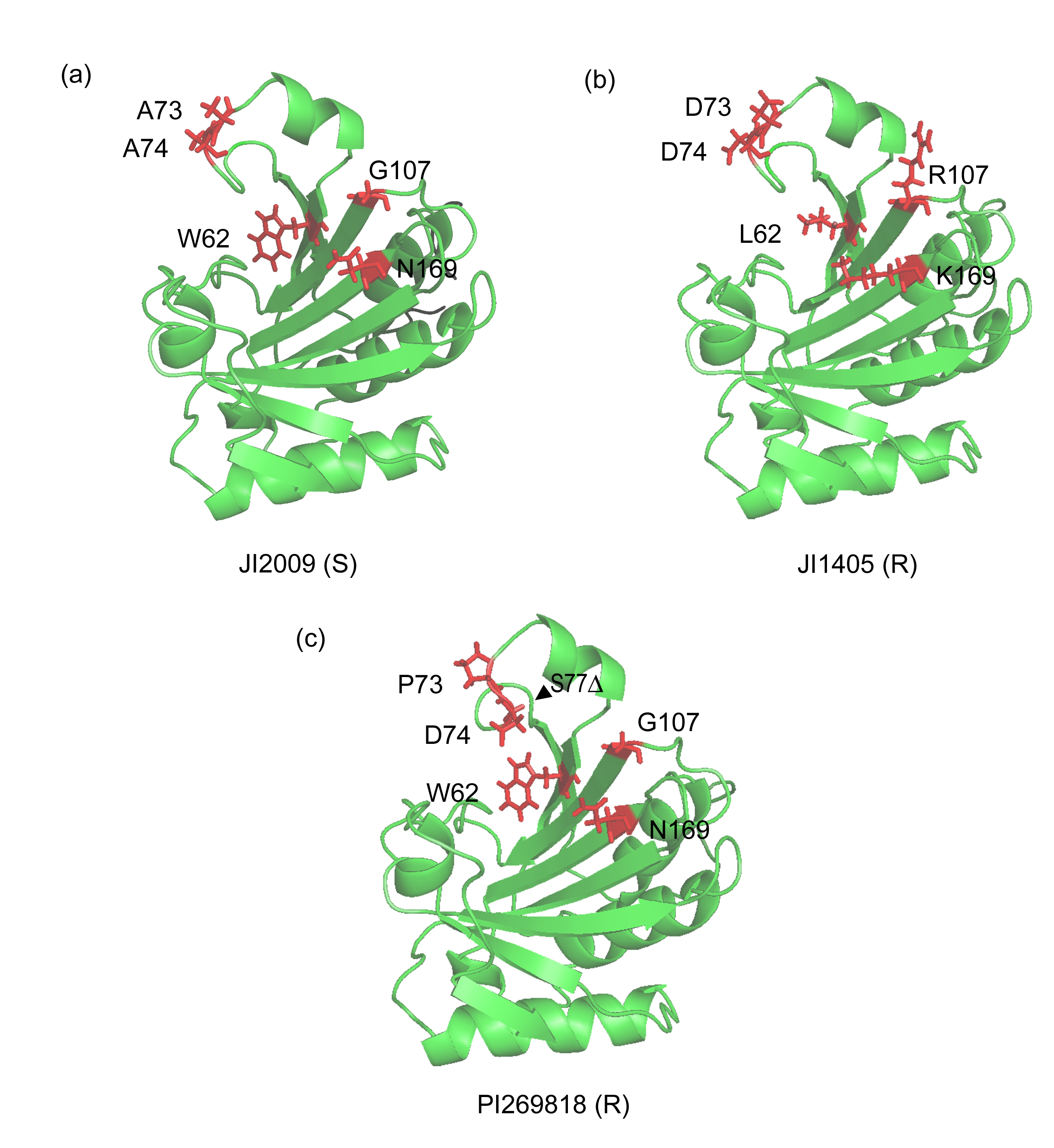

Supplement: Supplementary file 1 — Figure S1 Three‐dimensional predicted structure of Pisum sativum eIF4E proteins. [file PBI-16-1569-s005.tif]

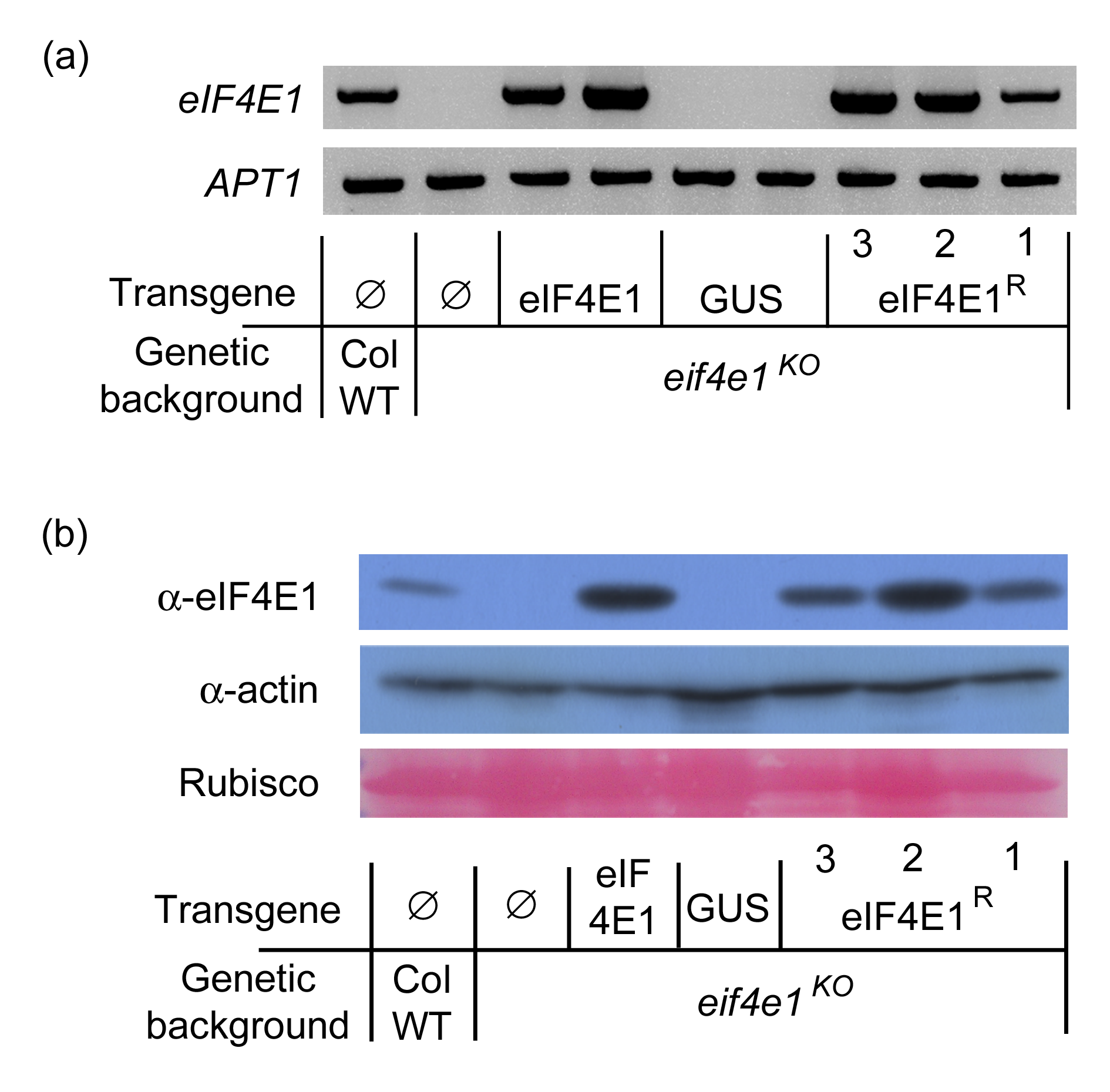

Supplement: Supplementary file 2 — Figure S2 eIF4E1 expression analysis in controls and complemented lines. [file PBI-16-1569-s008.tif]

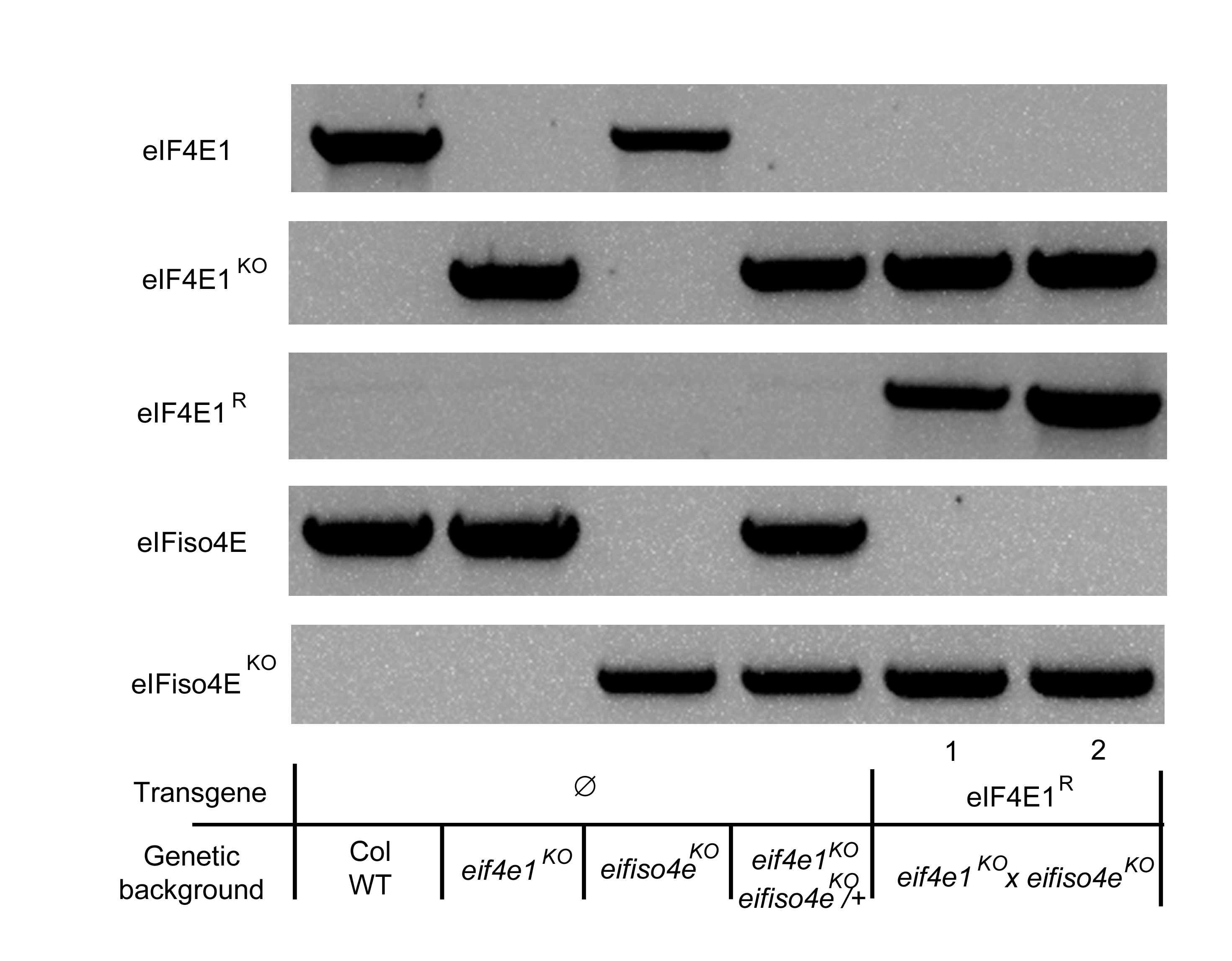

Supplement: Supplementary file 3 — Figure S3 Genotyping of eif4e1 KO eifiso4e KO eIF4E1 R plants. [file PBI-16-1569-s007.tif]

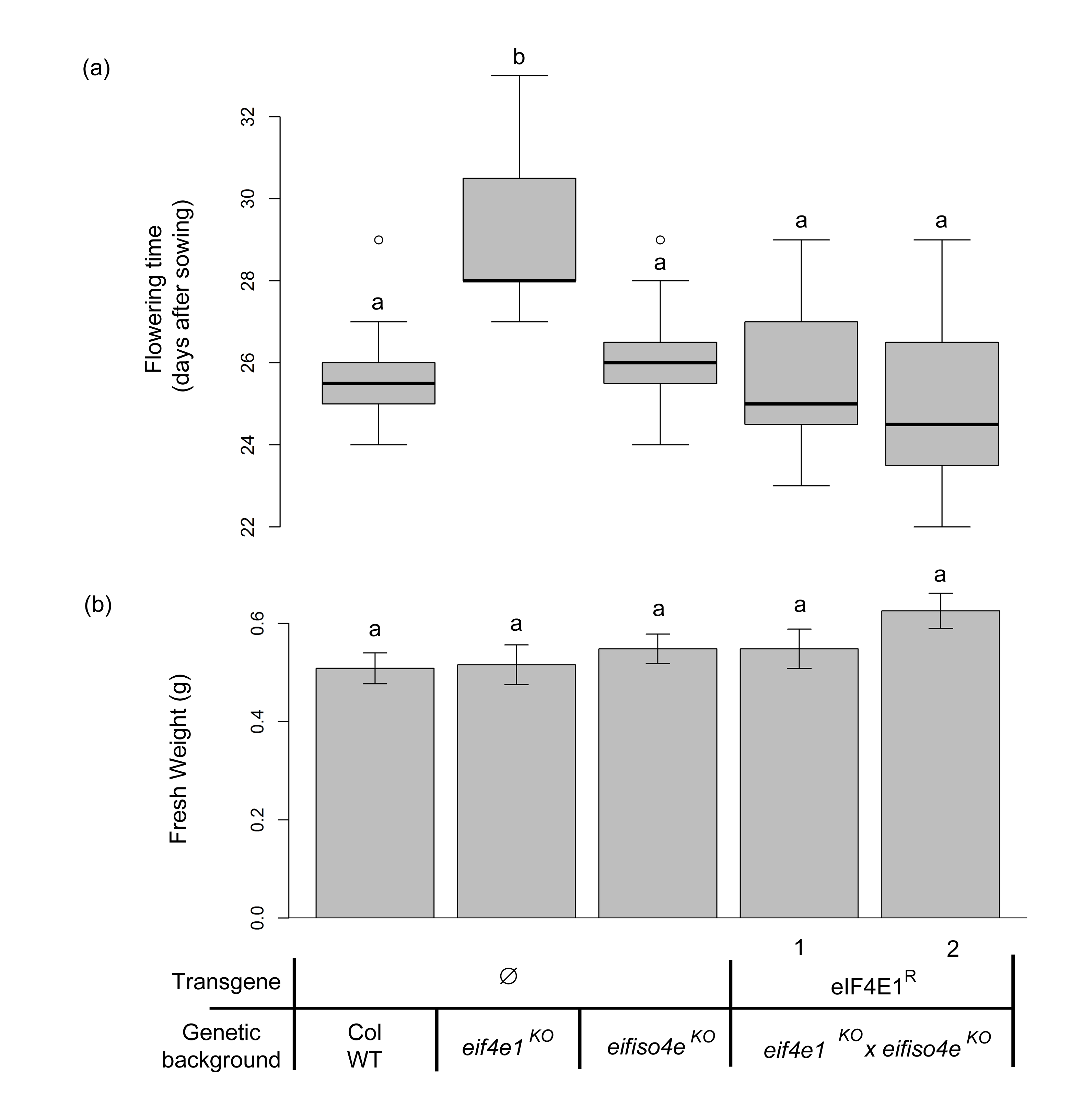

Supplement: Supplementary file 4 — Figure S4 Phenotype of the eif4e1 KO eifiso4e KO eIF4E1 R plants. [file PBI-16-1569-s009.tif]

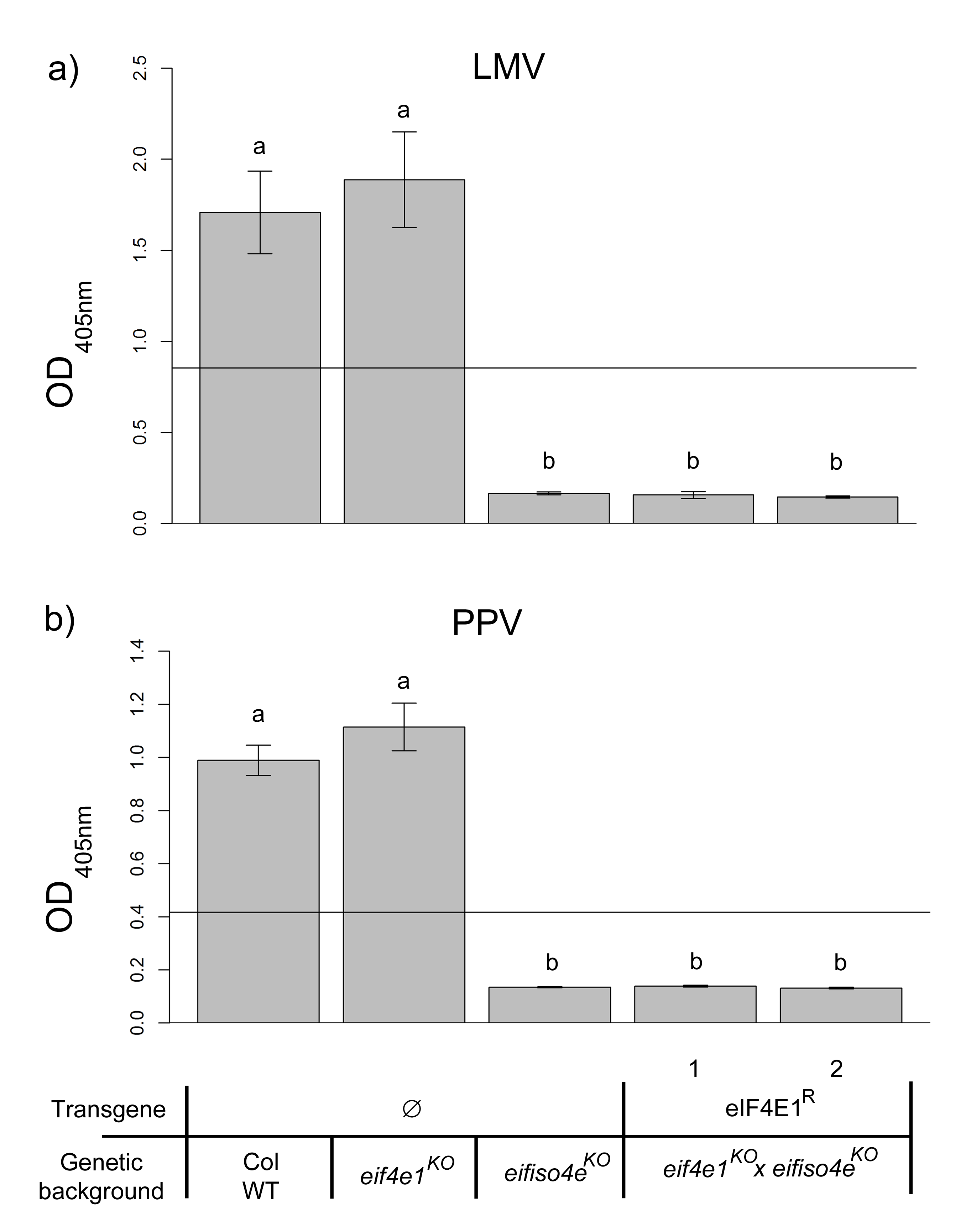

Supplement: Supplementary file 5 — Figure S5 eif4e1 KO eifiso4e KO eIF4E1 R plants resistance spectrum extends to LMV and PPV. [file PBI-16-1569-s001.tif]

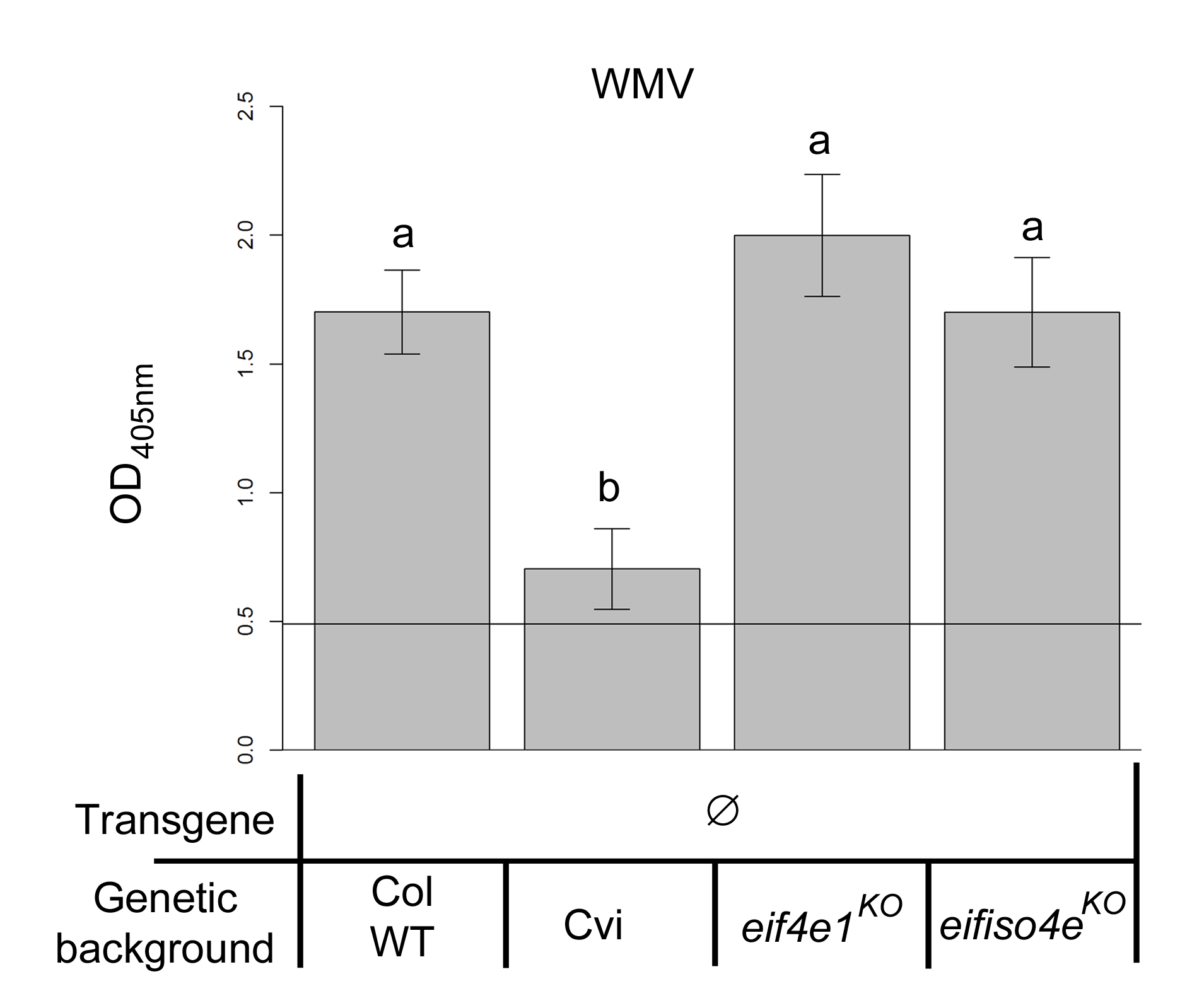

Supplement: Supplementary file 6 — Figure S6 The Cvi accession is partly resistant to WMV, but eif4e1 KO and eifiso4e KO single mutants are both susceptible. [file PBI-16-1569-s002.tif]

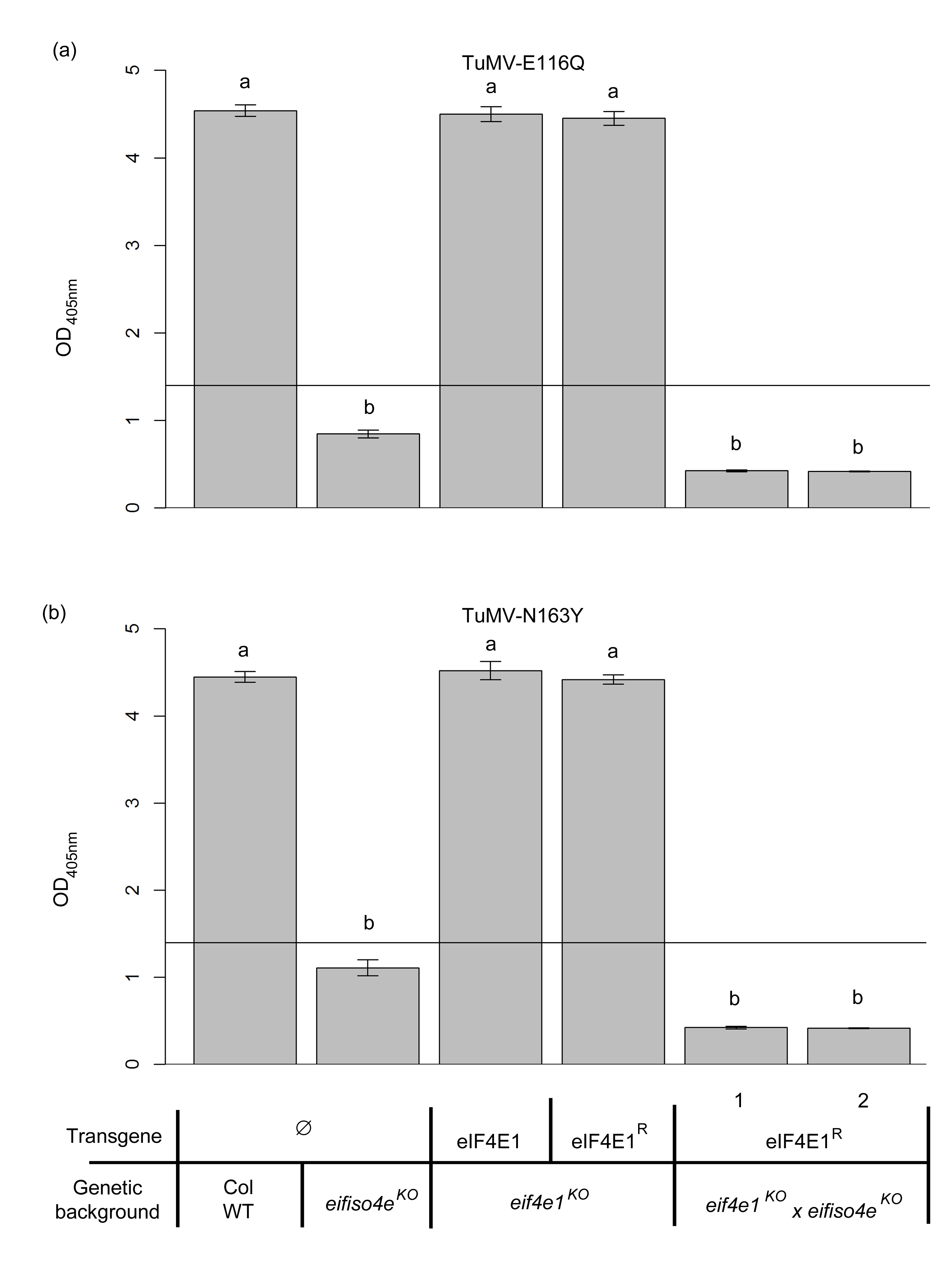

Supplement: Supplementary file 7 — Figure S7 Low accumulation of resistance‐breaking TuMV isolates in eifiso4e KO plants as detected by DAS‐ELISA [file PBI-16-1569-s003.tif]

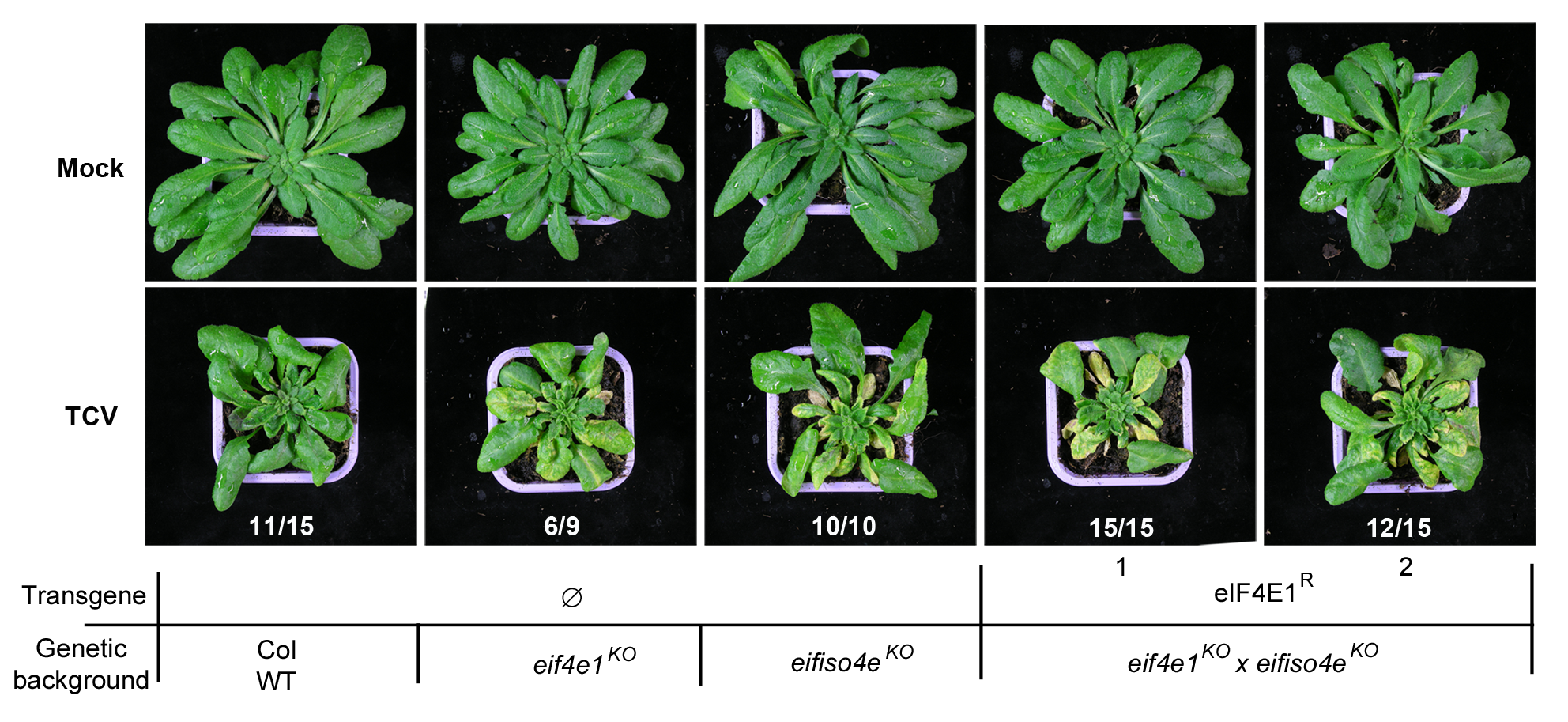

Supplement: Supplementary file 8 — Figure S8 eif4e1 KO eifiso4e KO eIF4E1 R plants resistance spectrum does not extend to carmovirus TCV. [file PBI-16-1569-s004.tif]

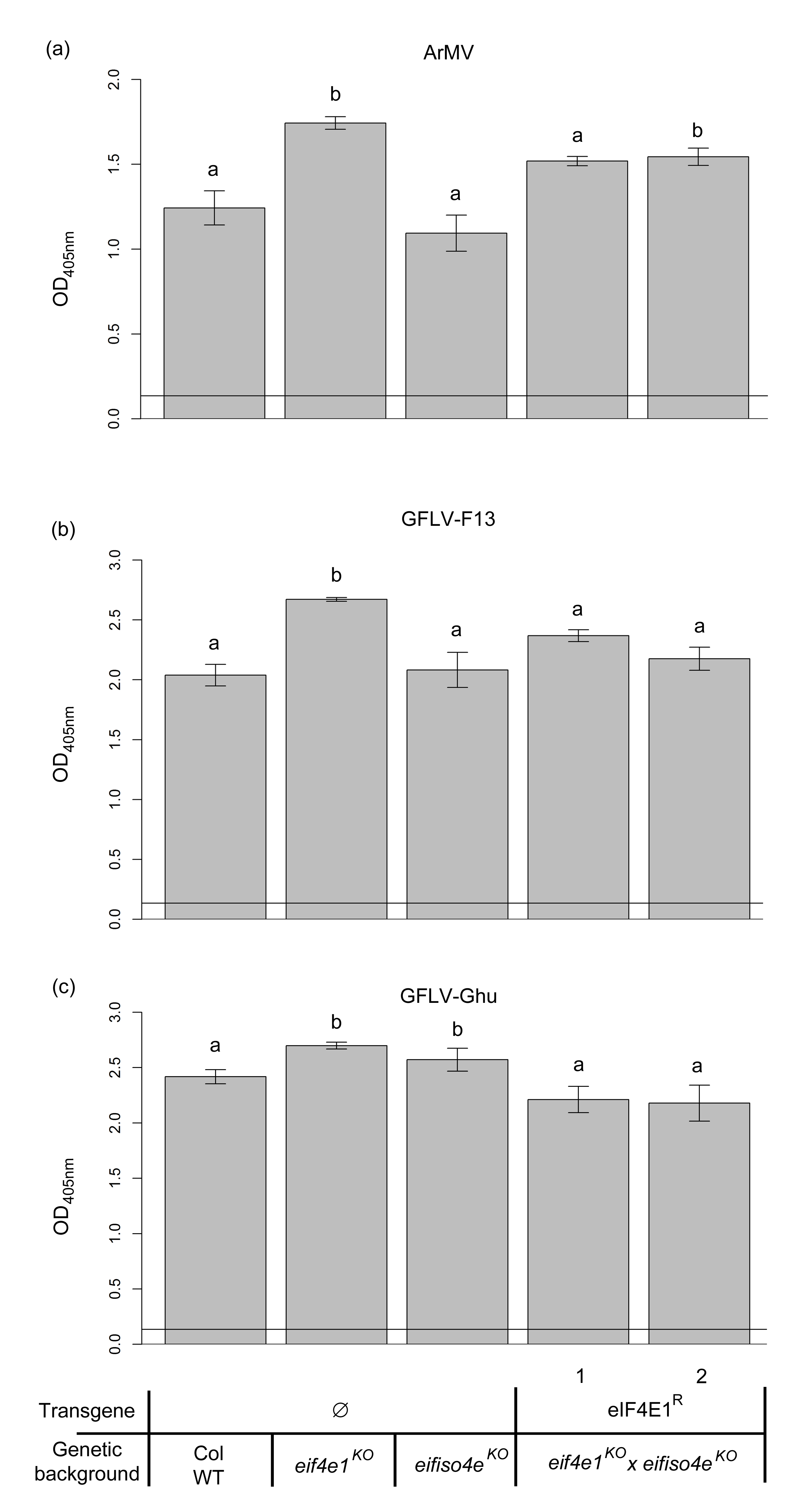

Supplement: Supplementary file 9 — Figure S9 eif4e1 KO eifiso4e KO eIF4E1 R plants resistance spectrum does not extend to nepoviruses. [file PBI-16-1569-s010.tif]
